# Design and synthesis of novel enantiopure Bis(5-Isoxazolidine) derivatives: insights into their antioxidant and antimicrobial potential via in silico drug-likeness, pharmacokinetic, medicinal chemistry properties, and molecular docking studies

**DOI:** 10.1016/j.heliyon.2022.e09746

**Published:** 2022-06-24

**Authors:** Arwa AL-Adhreai, Mohammed ALSaeedy, Ali Alrabie, Inas Al-Qadsy, Sam Dawbaa, ZabnAllah M. Alaizeri, Hisham A. Alhadlaq, Abdulrahman Al-Kubati, Maqusood Ahamed, Mazahar Farooqui

**Affiliations:** a Department of Chemistry, Maulana Azad of Arts, Science and Commerce, Aurangabad, 431004, India; b Department of Chemistry, Faculty of Science, Anadolu University, 26470, Eskişehir, Turkey; c Department of Pharmaceutical Chemistry, Faculty of Pharmacy, Anadolu University, 26470, Eskişehir, Turkey; d Department of Physics and Astronomy, College of Science, King Saud University, Riyadh, 11451, Saudi Arabia; e Department of Chemistry "Giacomo Ciamician" University of Bologna, 40126, Bologna, BO, Italy

**Keywords:** Isoxazolidines, Bicycloadducts, Nitrones, 1,3-Dipolar cycloaddition reaction., Drug-likeness, Docking study

## Abstract

A series of novel compounds, mono-5-isoxazolidines, and bis (5-isoxazolidines) derivatives, were prepared as bicycloadducts. The new series of isoxazolidines were designed and synthesized via 1,3-dipolar cycloaddition reaction of nitrones with 3,9-Divinyl-2,4,8,10-tetra oxaspiro (5-5) undecane in the context of new antimicrobial and antioxidant drugs discovery and were fully characterized by FT-IR, ^13^C-NMR, and ^1^H-NMR spectroscopy. The physicochemical properties of all the novel cycloadducts, like bioactivity score and lipophilicity, were predicted using calculative methods. Similarly, the pharmacokinetic properties such as metabolism, absorption, distribution, and excretion (ADME) were also predicted. Most of the tested compounds exhibited antimicrobial properties to varying degrees against various bacterial species, including the Gram-negative bacteria *Pseudomonas aeruginosa* and *Escherichia coli*, and the Gram-positive bacteria *Streptococcus pyogenus* and *Staphylococcus aureus*, Antifungal properties were also observed against the tested fungi like *Candida albicans*, *Aspergillus niger*, and *Aspergillus clavatus*. The activity data exhibited that most compounds have high activity as compared to the standard drugs. In the range of graded doses, the results of some selected compounds revealed that some are high antioxidants while others are moderate or weak antioxidants. As evidenced by the molecular docking studies, the synthesized compounds showed good binding mode better than a standard drug, against the protein of a Pantothenate Synthetase enzyme (PDB-2X3F).

## 1 Introduction

Currently, the progressive increase in infections induced by increasing the bacterial and fungal resistance against the antimicrobials owing to the widespread antibiotic use is the major cause of disease and even death due to therapeutic effectiveness being substantially reduced [1]. To combat microbial resistance and the emergence of new strains, we must develop new potent alternatives to potent antibacterial and antifungal agents with novel scaffolds [2, 3]. We observed that a lot of chemical families, such as isoxazolidines, are competent for these activities [4]. Isoxazolidine compounds possess several biological activities, such as antifungal, antibacterial [5], anticancer, and anti-inflammatory activities [6]. Nitrones, among the many 1,3-dipoles, are desirable as precursors because they may be easily [3 + 2] cycloaddition with alkenes to produce enantiopure chemical compounds with significant biological activities, such as heterocyclic isoxazolidines [7, 8] which can directly transform to into other biologically significant molecules such as amino sugars [9], β-lactams, alkaloids [10, 11], and amino acids [12] through reductive cleavage at the N–O bond [13]. Because of the ease with which this ring can be accessed via the 1,3- dipolar cycloaddition method [14], this heterocycle is particularly well suited for the synthesis of molecules useful in the design of new modified drugs.

(3 + 2) Cycloaddition of nitrones is among the most widely studied reactions. The formation of a new carbon-carbon bond and a carbon-oxygen bond at the same time, and the addition of groups rich or poor with electrons to the alkene leads to the polarization of the molecular orbitals of the C=C double bond in an alkene. The polarization of the molecular orbitals of the double bond in turn on the molecular orbitals symmetry of the substrates in the cycloaddition reactions in the transition state. This polarization affects the interaction between the highest occupied molecular orbital HOMO_Dipole_ – lowest unoccupied molecular orbital LUMO _Dipolarophile_ or LUMO _Dipole_–HOMO _Dipolarophile_ and thus controls the direction of how combined dipole and dipolarophile is. As a result, if the dipolarophile is monosubstituted, the high stereoselectivity leads to the generation of either 4-isoxazolidines or 5-isoxazolidines depending on the electronic character of the substituents. Most 5-substituted isoxazolidines are produced when using electron-rich dipolarophile, whereas in the instance of dipolarophile substituted with one group of an electron-withdrawing groups like –CN, –CHO, –CO_2_R lead to mostly the formation of 4-substituted isoxazolidines [8]. Thus, this can direct the cycloaddition process in either the Exo or endo transition states. Domingo [15] reported stereo and regioselectivity, which were dependent on the size of reactants as well.

Using simple nitrones leads to an endo-stereoselectivity, whereas the use of large dipolarophiles and nitrones direct the reaction to an Exo-stereoselectivity. Bis (isoxazolidines) are bis-adducts having two isoxazolidine rings. They are also symmetrical with respect to the center [16]. Bis (isoxazolidines) are synthesized via 1,3-dipolar cycloaddition of various nitrones with symmetric dipolarophiles containing two double bonds. Moreover, they have many industrial applications, including the production of pharmaceuticals, agrochemicals, or polymers [17]. Moreover, their well-known uses in the family of regular isoxazolidines as antibacterials and antifungals, etc.

**Scheme 1:**
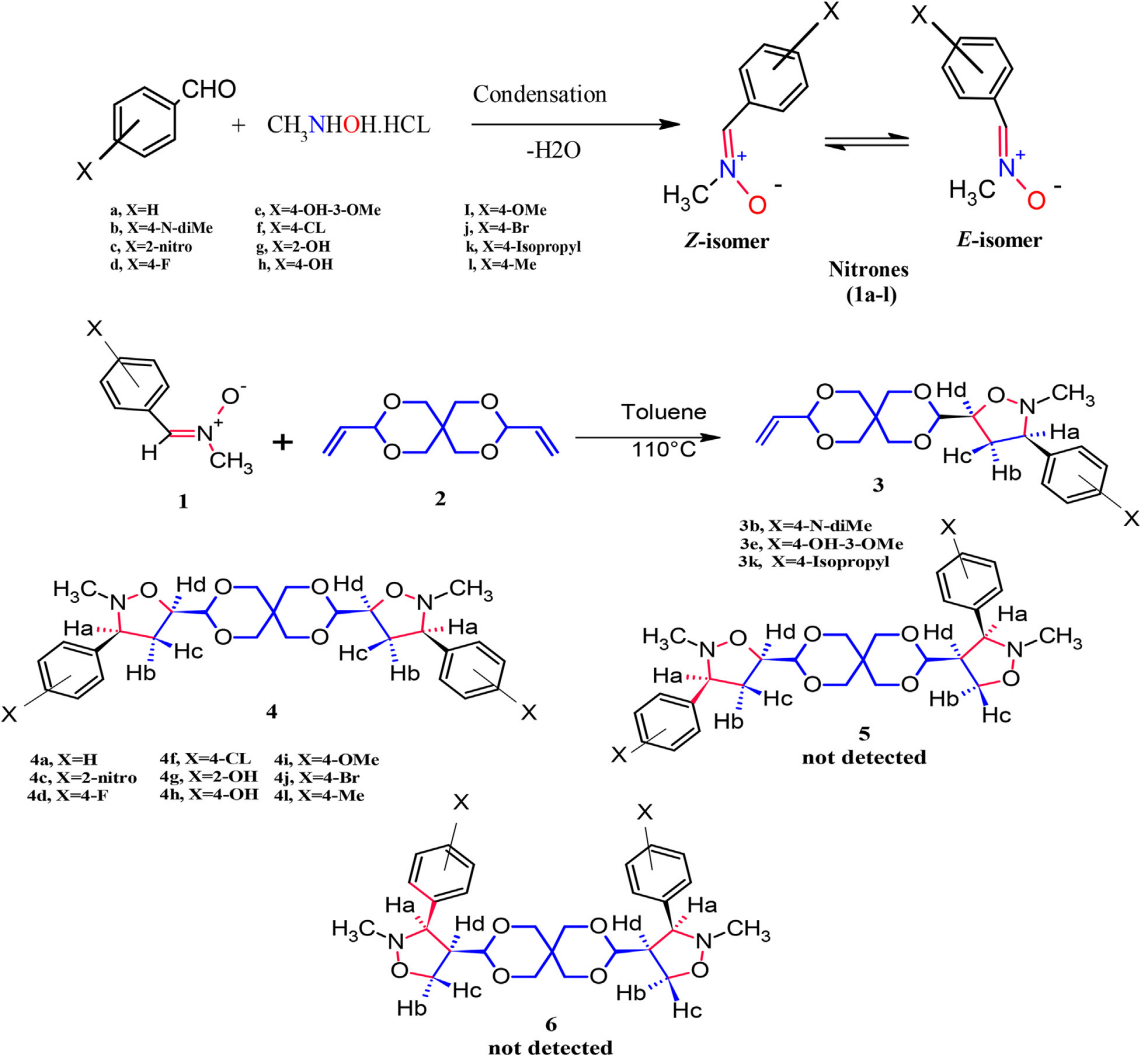
Synthesis of nitrone**(1a-j)** and of isoxazolidines derivatives. (**1**) Nitrones (1a-l); **(2)** bis-dipolarophile; **(3)** mono-5-isoxazolidines isomer**; (4)** (bis-5 isoxazolidine isomer); **(5)** (bis-4,5- isoxazolidine isomer); **(6)** (bis-4 isoxazolidine isomer).

In this study, we designed, synthesized, and identified a novel series of mono -5-isoxazolidines, bis (5-isoxazolidines) **3**, and **4**, respectively, via 1,3-dipolar cycloaddition for C-aryl-N-methyl nitrones (**1a-l**) with the symmetric alkene (3,9-Divinyl-2,4,8,10-tetra oxaspiro (5-5) (see Scheme 1). In order to continue drug discovery and development research, we have dedicated our efforts to the synthesis of bioactive heterocyclic compounds that can be used as powerful antioxidants and antibacterial agents. Furthermore, the regio and stereoselectivity were thoroughly discussed. *In vitro,* the antibacterial and antioxidant properties of these isoxazolidines were investigated. To predict their pharmacokinetic and pharmacodynamic characteristics, Using SwissADME online software, the targeted compounds were subjected to in silico evaluation (ADME). Furthermore, molecular docking studies were carried out for the most active analogs to explore their potential to become clinical drugs.

## 2 Materials and methods

### 2.1 Chemicals and physical measurements

All chemicals were obtained from Sigma Aldrich Company, and the purity was between 99%-99.9%. Thin-layer chromatography (TLC) was used to monitor the reaction's completion, with benzene and methanol mixture (8:2 volume ratio) as the mobile phase. CL-726 digital equipment (IndiaMART Member Since, Noida, India) was used to measure the melting points. A Nicolet iS10 spectrophotometer (Thermo Scientific, Materials 2020, 13, 514 3 of 16 Waltham, MA, USA) with an attenuated total reflection (ATR; diamond crystal) accessory was used for FTIR studies. With a total of 32 scans per spectrum and a spectral resolution of 4 Cm^-1^, all scans were performed in the 4000–500 cm^−1^ range. A JEOL ECP400 NMR spectroscope (Tokyo, Japan) was used to record ^1^H and ^13^C-NMR spectra at 400 MHz. Elemental microanalysis of the compounds was determined using the EA 300 (C.H.N.S) Element analyzer.

### 2.2 Physicochemical and ADME properties

SwissADME (http://www.swissadme.ch/) [3, 18, 19, 20] is a web tool that is utilized to compute the physicochemical characteristics as well as to predict the pharmacokinetic properties, ADME parameters. Besides, it also helps to predict the druglike nature and medicinal chemistry of compounds to support drugs discovery. The synthesized isoxazolidines' Physicochemical Properties were studied, such as: **(1)** Molecular weight 150 g/mol< MW > 500 g/mol; **(2)** Hydrogen Bond Acceptors < 10; **(3)** 0 < number of rotatable bonds < 9; **(4)** Hydrogen Bond Donors <5; **(5)** INSATU: 0.25 < fraction of Csp3 < 1; **(6)** the polarity: 20 Å^2^ < topological surface area (TPSA) < 160 Å^2^. The pharmacokinetics and drug-likeness properties of the synthesized drugs, such as blood-brain barrier permeation (BBB), human gastrointestinal absorption (GI), skin permeability parameter, plasma P-glycoprotein protein binding (P-gp), and interaction of the synthesized compounds with five important enzymes of the human cytochromes(P450) CYP1A2, CYP2D6, CYP2C19, CYP3A4, and CYP2C9 (the enzymes responsible for 90% of drug metabolism) [21] as well as interfering with the metabolism of a variety of endogenous substances. the drug-likeness features and medicinal chemistry are presented in Table 3 for all derivatives. In addition, the bioavailability radar was performed by observing the pink area in the plot, which reflects the optimal range of each characteristic. If the molecule's radar plot's pink zone is totally in the red zone, it means the molecule possesses drug-like properties [22]. The Boiled-Egg model was also used to evaluate pharmacokinetics (see Figure 6).

### 2.3 Antimicrobial assay

Microbes that were used for antimicrobial activity evaluation were: first, bacterial strains were the Gram-positive bacteria *Streptococcus pyogenes (MTCC 442)* and *Staphylococcus aureus (MTCC 96)* as well as the Gram-negative bacteria *Pseudomonas aeruginosa (MTCC 1688)* and *Escherichia coli (MTCC 443)*; second, fungal strains were *Aspergillus niger (MTCC 282)*, *Aspergillus clavatus (MTCC 1323),* and *Candida albicans (MTCC 227)*. The strains were generated at Chandigarh's Institute of Microbial Technology. A minimum inhibitory concentration (MIC) test was performed using the macro double dilution method [23]. To obtain the required concentration of the selected compounds to test on standard bacterial strains, DMSO was employed as a solvent with concentration 5%. In this screening, 1000, 500, 250, 200, 100, 50, 25, 12.5, and 6.25 μg/ml concentrations of the synthesized compounds were prepared and put in marked test tubes. To each, a tube was added of 1 mL of nutrient broth and 10 μL of fungi or bacteria strains, respectively. All the tubes were incubated for 18–24 h at 37 °C. Finally, they were observed for turbidity or growth and were compared to the reference drugs. Ampicillin and Chloramphenicol that have been selected as a standard for the antibacterial drug because they are a broad-spectrum antibiotic, are used to treat infections by many Gram-positive and Gram-negative bacteria, also Nystatin and Greseofulvin have been selected as reference antifungal drugs. They are an antifungal medicine. They are used to treat or prevent infections caused by a fungus (or yeast).

### 2.4 Antioxidant assay

A Schimadzu Double Beam UV-Visible Spectrophotometer was used to measure the required absorbance, (2 mM) of 2,2-diphenyl-1-picrylhydrazyl (DPPH) was dissolved in 95% ethanol. Similarly, the test samples were prepared in 95% ethanol. The blank was 2 mL of 95% ethanol. The measurement sample was prepared by mixing 1ml of DPPH with 1ml of the sample. Absorbance was carried out in the 515 nm range. The ascorbic acid solution was employed as a reference antioxidant (positive control) for comparison, and all measurements were recorded in triplicate readings. The samples were evaluated at 8 different concentrations (1, 2, 5, 10, 25, 50, 100, and 200 μg/mL), and the IC_50_ values were calculated based on the dose-response curve and are presented in Table 3.

### 2.5 Molecular docking

Isoxazolidine derivatives have been found to exhibit antibacterial activity in a review of the literature. Methicillin-resistant *Staphylococcus aureus* (MRSA) has become a main public health pother. MRSA is known for its multidrug resistance [24]. The 3D Crystal Structure of the Methicillin-Resistant *Staphylococcus aureus* Sar2676 (PDB code: 2X3F) protein [25]that has not undergone any mutation in the amino sequence, with a resolution of 1.95 Å was retrieved from the protein data bank (http://www.rscb.org) and the molecular docking was performed using Molecular Operating Environment (MOE 2015.10) software.

#### 2.5.1 Pre-docking preparation

Both synthesized compounds (ligands) and protein were prepared, minimized, and optimized using MOE software. By using the builder module of MOE, the 3D structures of ligands were built, 3D-protonated, partial charge was applied, energy was minimized by applying MMFF94x force field with RMS gradient of 0.05 kcal/mol/A2, and prepared ligands were saved as a database file in the MOE form. The target structure (PDB: 2X3F) was prepared following the detailed procedures described earlier [26], water molecules, B chain, and SO4 were deleted, 3D protonation was done, energy minimization was applied, and 4464 atoms were corrected. The active site of protein was assigned using the MOE software's site finder module.

#### 2.5.2 Docking methodology

The methodology used for docking was similar to that previously described [27], The Tringle Matcher algorithm, London dG, was applied with MOE docking parameters (function 1). During the refinement, the receptor was kept rigid while the ligands were permitted to be flexible. Each molecule was permitted to interact with the protein in five different interactions. The best-fitted poses' docking scores with the active site at (2 × 3F) were then recorded.

### 2.6 General procedure for the preparation of C-aryl-N- methyl nitrones(1a-l)

Nitrones were prepared according to ref. [28] with slight modification. N-methylhydroxylaminehydrochloride (750mg, 9mmol) was dissolved in absolute ethanol (20ml), the mixture was stirred and warmed to 40 °C, and (10ml) of absolute ethanol was added slowly for completing the dissolution of the N-methylhydroxyl amine hydrochloride. Then sodium acetate (737mg, 9m mol) was added to neutralize the hydrochloride. To this stirred solution, a substituted benzaldehyde was added dropwise. The resulting mixture was stirred magnetically overnight at room temperature in the dark for (24h). When TLC shows completion of the reaction, the crude nitrone product was filtered, dried over anhydrous MgSO_4_, evaporated, and recrystallized by hot benzene.

#### 2.6.1 Preparation of C-(phenyl)-N-methylnitrone(1a)

Benzaldehyde (913mg) was used for the synthesis as described in the general procedure. Yield 80%; M.p.80–82 °C; R_f_ 0.45, IR(KBr): 3389 (aromat. H), 3061 (CH_3_), 1695(C=N),1594-1442(aromatic.C=C), 1158 (N^+^-O^-^),1096(C–N), 838, 781, 687 (Para-disubstituted benzene) Cm^−1^. ^1^H-NMR (400 MHz, CDCl_3_): δ (ppm) = 8.00–7.37 (m,5H, aromat. H),7.15 (CH = N) 3.76 (s, 3H, N–CH_3_), ^13^C-NMR (400 MHz, CDCl_3_): δ (ppm) = 55.00 (N–CH_3_), 128.57–132.00, (Aromat. C), 134.0 (CH = N).

#### 2.6.2 Preparation of C-(4- N-Di methylphenyl)-N-methylnitrone(1b)

4- N-Dimethylamine benzaldehyde (1342mg) was used for the synthesis as described in the general procedure. Yield 72%; M.p.108–110 °C; R_f_ 0.72, IR(KBr): 3347(N–CH_3_),3086(aromat. H),2889 (CH_3_),1595 (C=N),1516–1435 (aromatic. C=C), 1178(N^+^-O^-^), 1065(C–N), 887, 808 (Para-disubstituted benzene) Cm^−1^. ^1^H-NMR (400 MHz, CDCl_3_): δ (ppm) = 8.22–7.22 (m,5H, aromat.H),7.25 (CH = N), 3.87 (s, 3H, N–CH_3_), 3.21 (s, 6H, N–CH_3_),^13^C-NMR (400 MHz, CDCl_3_): δ (ppm) = 39.87(H_3_C–N–CH_3_), 56.65 (N–CH_3_), 120.44–145.06, (Aromat. C), 134.32 (CH = N).

#### 2.6.3 Preparation of C-(2- nitrophenyl)-N-methylnitrone(1c)

2- Nitrobenzaldehyde (1360 mg) was used for the synthesis as described in the general procedure. Yield 84%; M.p.84–86 °C; R_f_ 0.60, IR(KBr): 3095(aromat. H),2976(CH_3_), 1698(C=N), 1574(NO_2_), 1507-1414 (aromatic. C=C), 1169 (N^+^-O^-^), 1054 (C–N), 935, 734, 663 (Ortho-disubstituted benzene) Cm^−1^. ^1^H-NMR (400 MHz, CDCl_3_): δ (ppm) = 8.02–7.20 (m,4H, aromat.H), 7.15 (CH = N), 3.72 (s, 3H, N–CH_3_). ^13^C-NMR (400 MHz, CDCl_3_): δ (ppm) = 56.33 (CH_3_), 126.00–150.00, (Aromat. C), 135.03 (CH = N).

#### 2.6.4 Preparation of C-(4- fluorophenyl)-N-methylnitrone(1d)

4- Florobenzaldehyde (1117 mg(was used for the synthesis as described in the general procedure. Yield 86%; M.p.99–102 °C; R_f_ 0.64, IR(KBr): 3015 (aromat. H), 2947(CH_3_), 1597 (C=N), 1504-1412(aromatic.C=C), 1230(C–F), 1154(N^+^-O^-^), 1048(C–N), 937, 834, 792 (Para-disubstituted benzene) Cm^−1^.^1^H-NMR (400 MHz, CDCl_3_): δ (ppm) = 3.75 (s, 3H, N–CH_3_), 7.15 (CH = N),7.20–7.28 (m, 2H, aromat.H. a,e), 8.02–8.08 (m,2H,aromat.H.b,d). ^13^C-NMR (400 MHz, CDCl_3_): δ (ppm) = 56.65 (N–CH_3_), 120.44–145.06, (Aromat. C), 134.07 (CH = N).

#### 2.6.5 Preparation of C-(4- hydroxy-3-methoxyphenyl)-N-methyl *nitrone (1e)*

4- Hydroxy-3-methoxy benzaldehyde (1369mg (was used for the synthesis as described in the general procedure. Yield 90%; M.p.171–173 °C; R_f_ 0.33, IR(KBr): 3353(O–H), 3059(aromat. H),2959,2840 (CH_3_),1670(C=N),1585, 1459 (aromatic –C=C-),1282(C–O), 1197(N^+^-O^-^),1023(C–N),910,879 (Para-substitutedbenzene)724,814(meta sub.) Cm^−1^. ^1^H-NMR (400 MHz, CDCl_3_): δ (ppm) = 9.52 (s,1H, OH),7.37 (d,1H, aromat, Hd) 7.68 (d, 1H, aromat.He), 7.42 (s, 1H, aromat.Ha),7.15 (CH = N), 3.76 (S, 3H, N–CH_3_). ^13^C-NMR (400 MHz, CDCl_3_): δ (ppm) = 54.33(O–CH_3_), 56.00 (N–CH_3_), 120.05–152.23, (Aromat. C), 135.0 (CH = N)140,78(C–OH).

#### 2.6.6 Preparation of C-(4-chlorophenyl)-N-methylnitrone(1f)

4-Chloro benzaldehyde (1265mg) was used for the synthesis as described in the general procedure. Yield 69%; M.p.114–117 °C; R_f_ 0.58, IR(KBr):3001 (aromat. H),2759 (CH_3_), 1673(C=N),1584-1474(aromatic.C=C), 1158(N^+^-O^-^),1079(C–N), 939,849,819 (Para-disubstituted benzene) Cm^−1^. ^1^H-NMR (400 MHz, CDCl_3_): δ (ppm) = 8.15–8.20 (m, 2H, aromat.H. b,d), 7.35–7.40 (m, 2H, aromat.H. a,e),7.30 (CH = N),3.85–3.90 (s, 3H, N–CH_3_). ^13^C-NMR (400 MHz, CDCl_3_): δ (ppm) = 54.00 (N–CH_3_), 127.00–134.74, (Aromat. C), 134.00 (CH = N).

#### 2.6.7 Preparation of C-(2- hydroxyphenyl)-N-methylnitrone(1g)

2- Hydroxy benzaldehyde(1099mg) was used for the synthesis as described in the general procedure. Yield 65%; M.p.134–136 °C; R_f_ 0.39, IR(KBr): 3446(O–H),3039 (aromat. H),2876 (CH_3_),1604(C=N),1580-1458(aromatic.C=C),1265(C–O), 1148(N^+^-O^-^), 1084(C–N),942,774 (ortho-disubstituted benzene) Cm^−1^. ^1^H-NMR (400 MHz, CDCl_3_): δ (ppm) = 9.73(OH),7.27–7.01 (m,4H, aromat.H),7.20 (CH = N), 3.82 (s, 3H, N–CH_3_). ^13^C-NMR (400 MHz, CDCl_3_): δ (ppm) = 56.33 (N–CH_3_), 121.00–130.39, (Aromat. C), 135.24 (CH = N), 144.27(C–OH).

#### 2.6.8 Preparation of C-(4-hydroxyphenyl)-N-methylnitrone(1h)

4- Hydroxy benzaldehyde (1099mg) was used for the synthesis as described in the general procedure. Yield 64%; M.p.178–180 °C; R_f_ 0.37, IR(KBr): 3446(O–H),3039(aromat. H), 2876 (CH_3_), 1580(C=N),1516-1485(aromatic C=C), 1205 (C–O), 1149(N^+^-O^-^), 1084(C–N), 774 (Para-disubstituted benzene) Cm^−1^. ^1^H-NMR (400 MHz, CDCl_3_): δ (ppm) = 9.63(OH),7.29–7.33 (m,2H, aromat. H.a,e), 7.14–7.17 (m, 2H, aromat.H b,d), 7.18 (CH = N), 3.86- (s, 3H, N–CH_3_). ^13^C-NMR (400 MHz, CDCl_3_): δ (ppm) = 56.00 (N–CH_3_), 123.20–145.05, (Aromat. C), 134.68(CH = N), 145.05(C–OH).

#### 2.6.9 Preparation of C-(4-methoxyphenyl)-N-methylnitrone(1i)

4-Methoxy benzaldehyde (1225mg) was used for the synthesis as described in the general procedure. Yield 75%; M.p.60–63 °C; R_f_ 0.60, IR(KBr):3083 (aromat. H), 2946,2838 (CH_3_), 1680(C=N), 1599-1413(aromatic.C=C), 1251 (C–O), 1155 (N^+^-O^-^), 1022(C–N),939,833, 774 (Para-disubstituted benzene) Cm^−1^. ^1^H-NMR (400 MHz, CDCl_3_): δ (ppm) = 7.38–7.06 (m,4H, aromat.H),7.46 (CH = N), 3.94 (S, 3H, CH_3_) 3.86 (S, 3H, N–CH_3_), ^13^C-NMR (400 MHz, CDCl_3_): δ (ppm) = 54.7(-CH_3_)56.78 (N–CH_3_), 129.00–132.05, (Aromat. C), 134.12 (CH = N).

#### 2.6.10 Preparation of C-(4-bromophenyl)-N-methylnitrone(1j)

4-Bromo benzaldehyde (1665mg) was used for the synthesis as described in the general procedure. Yield 82%; M.p.124–126 °C; R_f_ 0.53, IR(KBr): 3373(aromat. H),3001 (CH_3_),1676(C=N),1581-1466(aromatic. C=C), 1160 (N^+^-O^-^), 1057(C–N),998,940,849 (Para-disubstituted benzene) Cm^−1^. ^1^H-NMR (400 MHz, CDCl_3_): δ (ppm) = 7.19–7.35 (m, 4H, aromat. H), 7.28 (CH = N), 3.83 (s, 3H, N– CH_3_). ^13^C-NMR (400 MHz, CDCl_3_): δ (ppm) = 56.86 (N–CH_3_), 123.10–152.06, (Aromat. C), 135.11 (CH = N).

#### 2.6.11 Preparation of C-(4- Isopropylphenyl)-N-methylnitrone(1k)

4- Isopropyl benzaldehyde (1333mg) was used for the synthesis as described in the general procedure. Yield 85%; M.p. syrup; R_f_ 0.37, IR(KBr): 3046(aromat. H),2940,2886 (CH_3_),1612(C=N),1580-1457(aromatic. C=C), 1160 (N^+^-O^-^), 1050(C–N), 876, 806 (Para-disubstituted benzene) Cm^−1^. ^1^H-NMR (400 MHz, CDCl_3_): δ (ppm) = 7.36–7.10 (m, 4H, aromat. H),7.10 (CH = N), 3.74 (s, 3H, N–CH_3_), 2.98–3.03 (m, 1H, CH),2.07 (d,6H, CH_3_). ^13^C-NMR (400 MHz, CDCl_3_): δ (ppm) = 25.84(-CH_3_),41.50(-CH_2_) 57.00 (N–CH_3_), 122.00–141.18, (Aromat. C), 134.28 (CH = N).

#### 2.6.12 Preparation of C-(4- methylphenyl)-N-methylnitrone(1l)

4- Methyl benzaldehyde (1081mg) was used for the synthesis as described in the general procedure. Yield 72%; M.p.106–109 °C; R_f_ 0.34, IR(KBr): 3001(aromat. H), 2993, 2945 (CH_3_),1605(C=N),1500-1406 (aromatic. C=C), 1153(N^+^-O^-^),1043 (C–N),934,834,757 (Para-disubstituted benzene) Cm^−1^.^1^H-NMR (400 MHz, CDCl_3_): δ (ppm) = 7.95–7.10 (m,4H, aromat.H), 7.09 (CH = N), 3.95 (s,3H, N–CH_3_), 2.06 (s, 3H, Ar-CH_3_). ^13^C-NMR (400 MHz, CDCl_3_): δ (ppm) = 24.00 (Ar-CH_3_), 54.00(N–CH_3_), 126.50,128.02 (Aromat. C), 135.06 (C=N).

### 2.7 Preparation of isoxazolidine derivatives 3,4 (a-l)

With minor modifications, the isoxazolidine derivatives were prepared according to ref. [29, 30]. In a(100ml) round bottom flask. The nitrone was dissolved in toluene(25ml) with stirring, 3,9-Divinyl-2,4,8,10-tetra oxaspiro(5-5)undecane (200mg, 0.94mmol) was added to the solution. The reaction mixture was heated under reflux at 110 °C for (21–72h). The mixture was checked by the TLC technique to know the reaction's endpoint. When TLC shows that the reaction is completed, the resulting mixture was cooled and the solvent was evaporated, treated with water (20ml) and chloroform (30ml) was added. The organic layer was separated, dried with anhydrous sodium sulfate, filtered, the solvent was evaporated and was recrystallized with hot ethanol.

#### 2.7.1 Preparation of 2-methyl-5-(9-(2-methyl-3-phenylisoxazolidin-4-yl)-1,5,7,11-tetraoxaspiro [5.5] undecan-3-yl)-3-phenylisoxazolidine *(4a)*

Nitrone **(1a)** (254mg,1.88mmol) was used for the synthesis as described in the general procedure. The mixture was stirred for (48hr); yield 88%; M.p.77–79 °C; R_f_ 0.49, IR(KBr): 3087 (CH_3_),1588-1435(aromatic. C=C),1248-1203(C–O), 1163(N–O),1024(C–N), 985,850,755 (Mono-disubstituted benzene) Cm^−1^. ^1^H-NMR (400 MHz, DMSO-*D*_6_) δ = 7.36–6.96 (m, 10H,H-aromatic), 5.75 (ddd, *J* = 15.4, 10.7, 4.3 Hz, 2H,Hd), 5.36 (d, *J* = 11.1, 2H,Hh), 5.28 (d, *J* = 11.3, 2H,Hf), 4.85 (d, *J* = 13.9, Hz, 2H,He), 4.28 (dd, *J* = 11.3, 2.5 Hz, 2H,Ha), 3.78 (s, 6H,2CH_3_), 3.65–3.59 (m, 4H,2Hg,2Hf), 3.52 (ddd, *J* = 11.6, 5.2, 2.3 Hz, 2H,Hb), 3.42 (ddd, *J* = 11.6,6.2, 4.3 Hz, 2H,Hc). ^13^C-NMR (400 MHz, DMSO-*D*_6_) δ = 138.81, 129.42, 129.37, 128.16, 127.16, 127.10 (Ar–C), 119.45 (C4,4^∖^), 111.28(C5,5^∖^), 100.45(C7,7^∖^), 69.96(C3,3^∖^), 69.45(C2,2∖), 53.45(C6,6^∖^), 41.23(2CH_3_), 32.35(C1). Anal calc for C_26_H_29_F_2_N_2_O_6_: C, 62.02; H, 5.81; N, 5.56. Found: C, 62.12; H, 5.92; N, 5.66.

#### 2.7.2 Preparation of 4-(5-(9-(3-(4-(dimethylamino) phenyl)-2-methyl *isoxazolidin-*4-yl*)-1,5,7,11-tetraoxaspiro [5.5] undecan-*3-yl*)-2-methylisoxazolidin-*3-yl*)-N, N-dimethylaniline(3b)*

Nitrone (**1b**) (335mg,1.88mmol) was used for the synthesis as described in the general procedure. The mixture was stirred for (30hrs); yield 76%; M.p.85–87 °C; Rf 0.76, IR(KBr): 3000(N–CH_3_),2950 (CH_3_),1555-1420(aromatic.C=C),1245, 1203(C–O), 1162(N–O), 1073, 1040(C–N),899,817,720 (Para-disubstituted benzene) Cm^−1^. ^1^H-NMR (400 MHz, DMSO-*D*_6_) δ = 8.05 (d, *J* = 8.7 Hz, 2H), 6.78 (m,1H,H3),6.67 (d, *J* = 9.2 Hz, 2H), 6.26(m, 2H, H2,H4), 6.20 (dd, *J* = 17.3, 6.4 Hz, 1H, H1) 5.74 (ddd, *J* = 15.5, 10.8, 4.5 Hz, 1H, Hd), 5.35 (d, *J* = 13.7 Hz 2H,Hh), 5.25 (d, *J* = 13.5 Hz, 2H,Hf), 4.86 (d, *J* = 13.6Hz, 1H,He), 4.27 (dd, *J* = 11.3, 2.4 Hz, 1H, Ha), 3.63 (s, 3H, N–CH_3_), 3.60 (m, 2H,Hg), 3.56 (m, 2H,Hf), 3.43 (dt, *J* = 11.5, 2.5 Hz, 1H,Hc), 3.40 (dt, *J* = 11.6,4.5 Hz, 1H,Hb), 3.33 (s, 3H, N–CH_3_), 3.33 (s, 3H, N–CH_3_). ^13^C-NMR (400 MHz, DMSO-*D*_6_) δ = 151.39, 135.42, 129.97, 119.60(ArC), 132.09(C9), 125.05(C10), 119.60(C8), 118.98(C4,4^∖^), 111.60(C5,5^∖^), 100.87(C7,7^∖^), 69.96(C3,3^∖^), 69.44(C2,2^∖^), 53.67(C6,6^∖^), 42.13(2CH_3_),40.16 (4CH_3_), 32.44(C1). Anal calc for C_21_H_30_N_2_O_5_: C, 64.60; H, 7.74; N, 7.17. Found: C, 64.69; H, 7.82; N, 7.25.

#### 2.7.3 Preparation of 2-methyl-5-(9-(2-methyl-3-(2-nitrophenyl) isoxazolidin-4-yl)-1,5,7,11-tetraoxaspiro [5.5] undecane-3-yl)-3-(2-nitrophenyl) isoxazolidine(4c)

Nitrone **(1c)** ((339mg,1.88mmol) was used for the synthesis as described in the general procedure. The mixture was stirred for (16hrs); yield 74%; M.p.133–135 °C; R_f_ 0.69, IR(KBr): 2954 (CH_3_),1607, 1575(NO_2_), 1523-1436 (aromatic C=C),1247, 1203(C–O), 1162(N–O),1072(C–N),935,899,720(orth-o-disubstituted benzene) Cm^−1^. ^1^H-NMR (400 MHz, DMSO-D6) δ = 8.40 (d, *J* = 7.9 Hz,2H, aromatic H), 8.00–7.63 (m,2H, aromatic-H),7.62–7.60 (m, 2H, aromatic H), 7.59 (t, *J* = 7.7 Hz, 2H, aromatic-H), 5.81–5.67 (m, 2H,Hd), 5.34 (d, *J* = 11.1 Hz, 2H,Hh), 5.21 (d, *J* = 11.4 Hz, 2H,Hf), 4.85 (m, 2H,He), 4.28 (m, 2H,Ha), 3.78 (s, 6H,2CH_3_), 3.58 (m, 4H, Hg,Hi), 3.45 (m, 2H,Hb), 3.33 (m, 2H,Hc). ^13^C-NMR (400 MHz, DMSO-*D*_6_) δ = 148.19, 135.40, 133.61, 130.74, 129.90, 124.76(ArC), 118.96(C4,4^∖^), 101.89(C5,5^∖^), 100.89(C7,7^∖^) 69.96(C3,3^∖^), 69.45(C2,2^∖^), 54.62(C6,6^∖^), 44.09(2CH_3_), 32.44(C1). Anal calc for C_27_H_32_N_4_O_10_: C, 56.64; H, 5.63; N, 9.79. Found: C, 56.71; H, 5.70; N, 9.90.

#### 2.7.4 Preparation of 3-(4-fluorophenyl)-5-(9-(3-(4-fluorophenyl)-2-methylisoxazolidin-4-yl)-1, 5,7,11-tetraoxaspiro [5.5] undecan-3-yl)-2-methylisoxazolidine(4d)

Nitrone **(1d)** (288mg,1.88mmol) was used for the synthesis as described in the general procedure. The mixture was stirred for (17hrs); yield 87%; M.p. syrup; R_f_ 0.78, IR(KBr): 3085 (CH_3_),1504-1420(aromatic. C=C),1294-1247 (C–O), 1162(N–O),1048(C–N),937,834,792 (para-disubstituted benzene) Cm^−1^. ^1^H-NMR (400 MHz, DMSO-*D*_6_) δ = 8.32–8.24 (m, 4H,aromatic H), 7.27–7.18 (m, 4H,aromatic-H), 5.75 (ddd, *J* = 15.3, 10.4, 4.6 Hz, 2H,Hd), 5.34 (d, *J* = 11.6 Hz, 2H,Hh), 5.22 (d, *J* = 11.8 Hz, 2H,Hf), 4.86 (d, *J* = 13.8 Hz, 2H,He), 4.25 (dd, *J* = 11.3, 2.5 Hz, 2H,Ha), 3.72 (s, 6H,2CH_3_), 3.67–3 (m, 4H,2Hg,2Hf), 3.56 (ddd, *J* = 11.5, 5.1, 2.5 Hz, 2H,Hb), 3.35 (ddd, *J* = 11.8,6.5, 4.5 Hz, 2H, Hc). ^13^C-NMR (400 MHz, DMSO-*D*_6_) δ = 160.23, 139.32, 128.65, 127.98, (Ar–C), 119.13 (C4,4^∖^), 113.45 (C5,5^∖^), 100.82(C7,7^∖^), 69.98 (C3,3^∖^), 69.45(C2,2^∖^), 53.49(C6,6^∖^), 42.09(2CH_3_), 32.67(C1). Anal calc for C_26_H_29_F_2_N_2_O_6_: C, 62.02; H, 5.81; N, 5.56. Found: C, 62.09; H, 5.88; N, 5.64.

#### 2.7.5 Preparation of 4-(5-(9-(3-(4-hydroxy-3-methoxyphenyl)-2-methylisoxazolidin-4-yl)-1,5, 7, 11-tetraoxaspiro [5.5] undecan-3-yl)-2-methylisoxazolidin-3-yl)-2-methoxyphenol(3e)

Nitrone **(1e)** (340mg,1.88mmol) was used for the synthesis as described in the general procedure The mixture was stirred for (30hrs); yield 79%; M.p.124–126 °C; R_f_ 0.55, IR(KBr): 3079(OH),3028,2960 (CH_3_),1513-1436 (aromatic.C=C),1288, 1261,1249(C–O), 1161(N–O),1073(C–N),985,916(para-sub. benzene),720, 778 (meta-disubstituted benzene) Cm^−1^. ^1^H-NMR (400 MHz, DMSO-*D*_6_) δ = 9.5 (s,1H,OH), 8.08 (d, *J* = 1.8 Hz, 1H, aromatic H), 7.62 (s, 1H, aromatic H), 7.50 (dd, *J* = 8.3, 2.0 Hz, 1H, aromatic H),6.7 (m,1H,H3), 6.26(m, 2H, H2,H4), 6.20 (dd, *J* = 17.4, 6.2 Hz, 1H, H1) 5.75 (ddd, *J* = 15.2, 10.6, 3.9 Hz, 1H, Hd), 5.35 (d, *J* = 13.5 Hz 2H,Hh), 5.21 (d, *J* = 13.7 Hz, 2H, Hf), 4.86 (d, *J* = 13.5 Hz, 1H,He), 4.28 (dd, *J* = 11.4, 2.5 Hz, 1H, Ha), 3.72 (s, 3H, O–CH_3_)), 3.65 (m, 2H,Hg), 3.59 (m, 2H,Hi), 3.57 (s,3H, N–CH_3_),3.62 (dt, *J* = 11.4, 2.7 Hz, 1H,Hc), 3.42 (dt, *J* = 11.4,4.5 Hz, 1H,Hb). ^13^C-NMR (400 MHz, DMSO-*D*_6_) δ = 148.80, 135.41, 134.32, 123.49, 122.88, 119.87 (Ar–C), 132.45(C9),119.00(C10),118.60(C8), 115.74(C4,4^∖^), 111.97(C5,5^∖^), 100.87(C7,7^∖^) 69.95(C3,3^∖^), 69.43(C2,2^∖^), 55.75(C6,6^∖^)54.00(O–CH_3_), 42.68(N–CH_3_), 32.45(C1). Anal calc for C_20_H_27_NO_7_: C, 61.06; H, 6.92; N, 3.56. Found: C, 61.18; H, 7.01; N, 3.65.

#### 2.7.6 Preparation of 3-(4-chlorophenyl)-5-(9-(3-(4-chlorophenyl)-2-methylisoxazolidin-4-yl)-1,5,7,11-tetraoxaspiro [5.5] undecan-3-yl)-2-methylisoxazolidine(4f)

Nitrone **(1f)** (319mg,1.88mmol) was used for the synthesis as described in the general procedure. The mixture was stirred for (18hrs); yield 95%; m.p.101–103 °C; R_f_ 0.58, IR(KBr): 3031(CH_3_),1591-1435(aromatic.C=C),1285-1203(C–O), 1162(N–O),1073(C–N),935,838 (para-disubstituted benzene) Cm^−1^. ^1^H-NMR (400 MHz, DMSO-*D*_6_) δ = 8.24–8.02 (m, 4H, aromatic H),7.15–7.00 (m, 4H, aromatic-H), 5.71 (ddd, *J* = 15.1, 10.2, 4.1 Hz, 2H, Hd), 5.35 (d, *J* = 11.1, 2H, Hh), 5.24 (d, *J* = 11.3, 2H, Hf), 4.84 (d, *J* = 13.2, Hz, 2H, He), 4.28 (dd, *J* = 11.6, 2.2 Hz, 2H, Ha), 3.71 (s, 6H,2CH_3_), 3.63–3.58 (m, 4H,2Hg,2Hf), 3.54 (ddd, *J* = 11 .6, 5.5, 2.8 Hz, 2H, Hb), 3.32 (ddd, *J* = 11.9,6.3, 4.1 Hz, 2H,Hc). ^13^C-NMR (400 MHz, DMSO-*D*_6_) δ = 140.32, 138.96, 135.42, 129.97, (Ar–C), 118.60 (C4,4^∖^), 111.42(C5,5^∖^), 100.89(C7,7^∖^), 69.97(C3,3^∖^), 69.46(C2,2^∖^), 53.69(C6,6^∖^), 40.22(2CH_3_), 32.49(C1). Anal calc for C_27_H_32_Cl_2_N_2_O_6_: C, 58.81; H, 5.85; N, 5.08. Found: C, 58.87; H, 5.91; N, 5.14.

#### 2.7.7 Preparation of 2-(5-(9-(3-(2-hydroxyphenyl)-2-methylisoxazol*-idin-*4-yl*)-1,5,7,11-tetraoxaspiro [5.5] undecan-*3-yl*)-2-methylisoxa-zolidin-*3-yl*) phenol(4g)*

Nitrone (**1g**) (284mg,1.88mmol) was used for the synthesis as described in the general procedure. The mixture was stirred for (15hrs); yield 69%; m.p.112–114 °C; R_f_ 0.74, IR(KBr): 3036 (OH),2948(CH_3_),1584-1454(aromatic. C=C),1388,1259, 1203(C–O),1145(N–O),1072(C–N),932,812,765 (orthodisubstitutedbenzene) Cm^−1^. ^1^H-NMR (400 MHz, DMSO-D6) δ = 8.09 (s, 2H, OH), 7.41–7.28 (m, 4H,aromatic H), 6.86–6.74 (m, 4H,aromatic-H), 5.74 (ddd, *J* = 15.3, 10.3, 4.1 Hz, 2H,Hd), 5.39 (d, *J* = 11.4, 2H,Hh), 5.21 (d, *J* = 11.6 Hz, 2H,Hf), 4.86 (d, *J* = 13.8, Hz, 2H,He), 4.27 (dd, *J* = 11.2, 2.4 Hz, 2H,Ha), 3.77 (s, 6H,2CH_3_), 3.67 (m, 2H,Hg), 3.57 (ddd, *J* = 11.5,4.2, 2.4 Hz, 2H,Hb), 3.43 (ddd, *J* = 11.6,5.6,4.4 Hz, 2H,Hc). ^13^C-NMR (400 MHz, DMSO-*D*_6_) δ = 159.03 (C–OH), 141.33, 135.41, 133.90, 132.71, 132.60 (Ar–C), 119.23(C4,4^∖^), 117.79(C5,5^∖^), 100.89(C7,7^∖^), 69.96 (C3,3^∖^), 69.44 (C2,2^∖)^, 52.2.7 (C6,6^∖^), 39.46 (2CH_3_), 32.44(C1). Anal calc for C_27_H_34_N_2_O_8_: C, 63.02; H, 6.66; N, 5.44. Found: C, 63.20; H, 6.86; N, 5.62.

#### 2.7.8 Preparation of 4-(5-(9-(3-(4-hydroxyphenyl)-2-methylisoxazoli*-din-*4-yl*)-1,5,7,11-tetraoxaspiro [5.5] undecan-*3-yl*)-2-methylisoxaz* lidin-3-yl) phenol(4h)

Nitrone **(1h)** (284mg,1.88mmol) was used for the synthesis as described in the general procedure. The mixture was stirred for (20hrs); yield 82%; M.p. syrup; R_f_ 0.77, IR(KBr): 3246(OH), 2976(CH_3_),1508-1430(aromatic.C=C),1274, 12.45 (C–O), 1158(N–O),1077(C–N),933, 854, 774 (Para-disubstituted benzene) Cm^−1^. ^1^H-NMR (400 MHz, DMSO-D6) δ = 9.16 (s, 2H,OH), 7.84–7.64 (m, 4H,aromatic H), 7.38–7.12 (m, 4H,aromatic-H), 5.76 (ddd, *J* = 15.5, 10.2, 4.4 Hz, 2H,Hd), 5.35 (d, *J* = 11.7, 2H,Hh), 5.26 (d, *J* = 11.5 Hz, 2H,Hf), 4.87 (d, *J* = 13.8, Hz, 2H,He), 4.25 (dd, *J* = 11.1, 2.3 Hz, 2H,Ha), 3.75 (s, 6H,2CH_3_), 3.67–3.61 (m, 4H,2Hg,2Hf), 3.56 (ddd, *J* = 11.3, 4.6, 2.5 Hz, 2H,Hb), 3.42 (dd, *J* = 11.0,5.5 4.8 Hz, 2H,Hc). ^13^C-NMR (400 MHz, DMSO-*D*_6_) δ = 156.66 (C–OH), 140.12, 130.96, 129.81, (Ar–C), 118.03(C4,4^∖^), 117.27(C5,5^∖^), 100.36(C7,7^∖^), 69.95(C3,3^∖^), 69.43(C2,2^∖^), 53.25(C6,6^∖^), 40.48(2CH_3_), 32.51(C1). Anal calc for C_27_H_34_N_2_O_8_: C, 63.02; H, 6.66; N, 5.44. Found: C, 63.19; H, 6.80; N, 5.56.

#### 2.7.9 Preparation of 3-(4-methoxyphenyl)-5-(9-(3-(4-methoxyphenyl*)-2-methylisoxazolidin-*4-yl*)-1,5,7,11-tetraoxaspiro [5.5] undecan-*3-yl*)-2-methylisoxazolidine(4i)*

Nitrone **(1i)** (311mg,1.88mmol) was used for the synthesis as described in the general procedure. The mixture was stirred for (30hr); yield 65%; M.p. syrup; R_f_ 0.52, IR(KBr): 3395 (O–CH_3_)3089 (CH_3_),1507-1420(aromatic. C=C), 1319, 1252 (C–O), 1161 (N–O),1073 (C–N),936, 840, 719 (para-disubstituted benzene) Cm^−1^. ^1^H-NMR (400 MHz, DMSO-*D*_6_) δ = 7.37–7.20 (m, 4H, aromatic H), 6.88–6.76 (m, 4H, aromatic H) 5.73(ddd, *J* = 15.6, 10.5, 3.4 Hz, 1H, Hd), 5.32 (d, *J* = 13.5 Hz 2H,Hh), 5.22 (d, *J* = 13.4 Hz, 2H,Hf), 4.83 (d, *J* = 13.7 Hz, 1H,He), 4.24 (dd, *J* = 11.2, 2.6 Hz, 1H, Ha), 3.85 (s, 3H, O–CH_3_), 3.62 (m, 2H,Hg), 3.58 (m, 2H,Hf), 3.54 (s,3H, N–CH_3_),3.63 (dt, *J* = 11.4, 2.4 Hz, 1H,Hc), 3.43 (dt, *J* = 11.6,4.5 Hz, 1H,Hb). ^13^C-NMR (400 MHz, DMSO-*D*_6_) δ = 155.70, 135.67, 123.49, 122.88, (Ar–C), 117.74 (C4,4^∖^), 111.63(C5,5^∖^), 100.38(C7,7^∖^), 69.95(C3,3^∖^), 69.43(C2,2^∖^), 57.73(C6,6^∖^),56.00(O–CH_3_), 52.11 (N–CH_3_), 32.42(C1). Anal calc for C_29_H_38_N_2_O_8_: C, 64.19; H, 7.06; N, 5.16. Found: C, 64.24; H, 7.10; N, 5.21.

#### 2.7.10 Preparation of 3-(4-bromophenyl)-5-(9-(3-(4-bromophenyl)-2-methylisoxazolidin-4-yl)-1,5,7,11-tetraoxaspiro [5.5] undecan-3-yl)-2-methylisoxazolidine(4j)

Nitrone **(1j)** (402mg,1.88mmol) was used for the synthesis as described in the general procedure. The mixture was stirred for (24hrs); yield 94%; M.p. semi-solid; R_f_ 0.56***,*** IR(KBr): 3088 (CH_3_),1587-1435(aromatic. C=C),1330-1247(C–O), 1163(N–O), 1071(C–N),984,953,821 (para-disubstituted benzene) Cm^−1^. ^1^H-NMR (400 MHz, DMSO-*D*_6_) δ = 8.20–8.02 (m, 4H,aromatic H), 7.14–7.01 (m, 4H,aromatic-H), 5.72 (ddd, *J* = 15.1, 10.4, 4.2 Hz, 2H,Hd),5.3 5 (d, *J* = 11.2, 2H,Hh), 5.22 (d, *J* = 11.3 Hz, 2H,Hf), 4.86 (d, *J* = 13.3, Hz, 2H,He), 4.24 (dd, *J* = 11.5, 2.2 Hz,2H,Ha), 3.72 (s, 6H,2CH_3_), 3.65–3.59 (m, 4H,2Hg,2Hf), 3.55 (ddd *J* = 11.5, 5.4, 2.8 Hz, 2H,Hb), 3.35 (ddd, *J* = 11.7,6.1, 4.1 Hz, 2H,Hc). ^13^C-NMR (400 MHz, DMSO-*D*_6_) δ = 137.48, 132.34, 128.12, 122.98, (Ar–C), 117.12(C4,4^∖^), 105.35(C5,5^∖^), 102.74(C7,7^∖^), 69.97(C3,3^∖^), 69.42(C2,2^∖^), 52.49(C6,6^∖^), 42.01(2CH_3_), 32.62(C1). Anal calc for C_27_H_32_Br_2_N_2_O_6_: C, 50.64; H, 5.04; N, 4.37. Found: C, 50.72; H, 5.11; N, 4.44.

#### 2.7.11 Preparation of 3-(4-isopropylphenyl)-5-(9-(3-(4-isopropylph*-enyl)-2-methylisoxazolidin-*4-yl*)-1,5,7,11-tetraoxaspiro [5.5] unde-can-*3-yl*)-2-methylisoxazolidine(3k)*

Nitrone **(1k)** (333mg,1.88mmol) was used for the synthesis as described in the general procedure. The mixture was stirred for (26hrs); yield 78%; M.p. syrup; R_f_ 0.63, IR(KBr): 3373,2959 (CH_3_),1508-1417(aromatic.C=C),1286,1246(C–O), 1152(N–O),1060(C–N),984, 899, 847 (para-disubstituted benzene) Cm^−1^. ^1^H-NMR (400 MHz, DMSO-*D*_6_) δ = 7.19 (m, 2H, aromatic H), 7.30 (d, *J* = 8.7,1.8 Hz, 2H, aromatic H), 6.75 (m,1H,H3), 6.24(m, 2H, H2,H4), 6.19 (dd, *J* = 17.2, 6.5 Hz, 1H, H1), 5.72 (ddd, *J* = 15.5, 10.2, 3.9 Hz, 1H, Hd), 5.34 (d, *J* = 13.3 Hz 2H,Hh), 5.22 (d, *J* = 13.7 Hz, 2H,Hf), 4.87 (d, *J* = 13.6 Hz, 1H,He), 4.26 (dd, *J* = 11.1, 2.7 Hz, 1H, Ha), 3.53 (m, 1H, CH)), 3.69 (m, 2H,Hg), 3.62 (m, 2H,Hf), 3.59 (s,3H, N–CH_3_),3.60 (dt, *J* = 11.3, 2.6 Hz, 1H,Hc), 3.44 (dt, *J* = 11.6,4.3 Hz, 1H,Hb)2.77 (d, *J* = 6.8 Hz, 6H,2CH_3_). ^13^C-NMR (400 MHz, DMSO-*D*_6_) δ = 148.77, 138.20, 123.49, 122.88, 119.00 (Ar–C), 134.10(C9), 118.76 (C10), 117.60(C8), 111.50(C4,4^∖^), 109.66(C5,5^∖^), 101.04(C7,7^∖^) 69.9.5 (C3,3^∖^), 69.44(C2,2^∖^), 57.70(C6,6^∖^), 54.38(N–CH_3_), 34.68(C–H),32.42(C1) 29.08(CH_3_). Anal calc for C_22_H_31_NO_5_: C, 67.84; H, 8.02; N, 3.60; O, 20.54 Found: C, 67.98; H, 8.16; N, 3.74.

#### 2.7.12 Preparation of 2-methyl-5-(9-(2-methyl-3-(p-tolyl) isoxazole*-din-*4-yl*)-1,5,7,11-tetraoxaspiro [5.5] undecan-*3-yl*)-3-(p-tolyl) isoxazolidine(4l)*

Nitrone **(1l)** (280mg,1.88mmol) was used for the synthesis as described in the general procedure. The mixture was stirred for (35hrs); yield 95%; M.p.99–103 °C; R_f_ 0.60, IR(KBr): 2954, 2849(CH_3_),1510-1421(aromatic.C=C),1343, 1247(C–O), 1163(N–O),1072(C–N), 937 (para-disubstituted benzene) Cm^−1^. ^1^H-NMR (400 MHz, DMSO-*D*_6_) δ = 7.34–7.23 (m, 4H, aromatic H), 7.12–7.03 (m, 4H, aromatic H), 5.74 (ddd, *J* = 15.2, 10.6, 3.9 Hz, 1H, Hd), 5.36 (d, *J* = 13.5 Hz 2H,Hh), 5.23 (d, *J* = 13.7 Hz, 2H,Hf), 4.88 (d, *J* = 13.5Hz, 1H,He), 4.25 (dd, *J* = 11.4, 2.5 Hz, 1H, Ha), 3.65 (m, 2H,Hg), 3.59 (m, 2H,Hf), 3.56.(s,3H, N–CH_3_),3.62 (dt, *J* = 11.4, 2.7 Hz, 1H, Hc), 3.42 (dt, *J* = 11.4,4.5 Hz, 1H, Hb), 2.88 (s,3H, CH_3_). ^13^C-NMR (400 MHz, DMSO-*D*_6_) δ = 135.80, 134.41, 123.49, 122.88 (Ar–C), 113.44(C4,4^∖^), 110.45(C5,5^∖^), 102.12(C7,7^∖^) 69.97 (C3,3^∖^), 69.42(C2,2^∖^), 57.77(C6,6^∖^), 54.20(N–CH_3_), 32.45(C1), 26.13(CH_3_). Anal calc for C_29_H_38_N_2_O_6_: C, 68.21; H, 7.50; N, 5.49. Found: C, 68.27; H, 7.56; N, 5.56.

## 3 Result and discussion

### 3.1 Chemistry

Our study began with an investigation of the synthesis of C-aryl-N-methyl nitrones (**1a-l**) by condensation of benzaldehyde derivatives with N-methyl, hydroxylaminehydrochloride which was separated, purified, and characterized followed by the cycloaddition reaction to nitrones (2 Equiv.) of (**1a-l**) with (1 Equiv.) of the dipolarophile **2** (3,9-Divinyl-2,4,8,10-tetra oxaspiro (5-5) undecane) as bis-dipolarophile in dry toluene at 110 °C under reflux for 20–40 h yielded cycloadduct **3** and **4** in good yield for the synthesis of bicyclic isoxazolidine analogs as in Scheme 1. It was shown that the reactions proceeded regio- and diastereoselectively, resulting in good to excellent yields of isoxazolidine derivatives (**3b, 3e, 3k, and 4a-l**). The isoxazolidines that we obtained were characterized spectroscopically. They are novel compounds that have never been presented in a study before. Their biological activity was studied and proved to have excellent efficacy, as well as being studied as an oral drug and proving different results. The formation of the cycloadducts was established by ^1^H-NMR,^13^C-NMR, and IR spectroscopy.

### 3.2 Spectral analysis

#### 3.2.1 FTIR analysis

As it is known that the infrared spectrum of the nitrones Figures S1-S10 (supplementary file).shows two important bands, they appeared at specific at the ranges between (1500–1660 Cm^-1^), (1100–1196 Cm^-1^) indicating stretching vibrations of (C=N, N^+^-O^-^) groups, respectively. These bands are evidence of the formation of the nitrone. We used compound (**1g**) as a model nitrones Figure S7, which showed in IR the bands of the groups (C=N), (N^+^-O^-^) at (1604,1169 Cm^-1^), respectively. The IR spectra of isoxazolidine of (**4g**) Figure S17 showed new absorption bands at (1072-1145-1388 Cm^−1^) which characterize the (CN, NO, and CO) groups in the isoxazolidine ring, respectively. It has been noticed that the absence of the C = N peak for the nitrone spectrum indicates the creation of an isoxazolidine ring as shown in Figures S11-S21 (supplementary file).

#### 3.2.2 NMR calculations

**Scheme 2:**
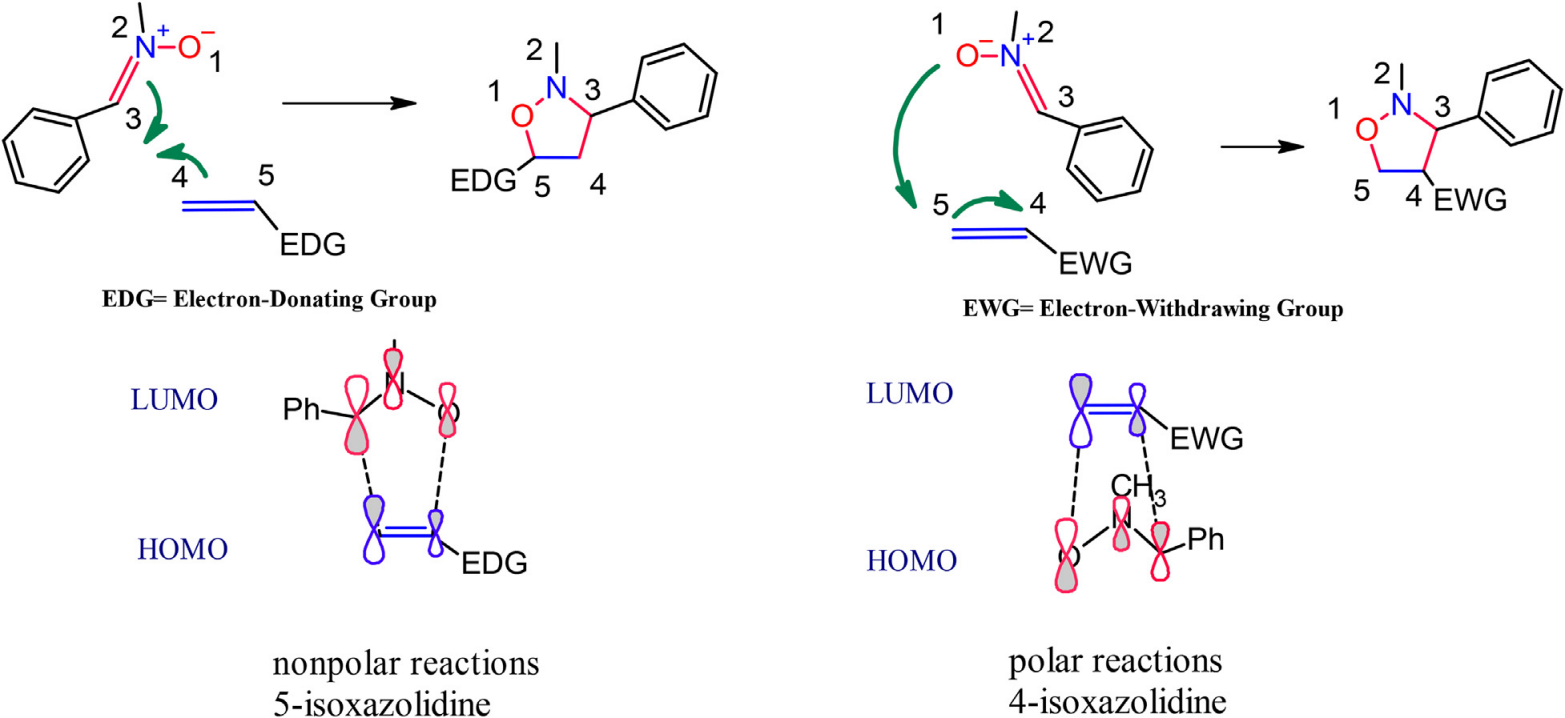
Regioselectivity of 1,3-dipolar cycloaddition.

To increase our understanding of the regio and stereochemical products of this cycloaddition reaction and the nature of the transition states involved, knowledge of the structure of the nitrones and alkenes was required. EZ isomerization has the potential to occur in acyclic nitrones. The H-NMR spectrum of nitrones Figures S22-S31 (supplementary file). showed that the yield of the reaction is a combination of isomers *E* and *Z* formed in a 5:1 isomerization equilibrium ratio, respectively. When we prepared the nitrones, we used a non-polar solvent, which is dichloromethane, and the solvent has a great effect on determining the dominant isomer, at these conditions, the dominant isomer is *E*, and the *Z*-isomer is dominant when using a polar solvent, such as a mixture of ethanol and water. When the reaction of the addition of a nitrone with an olefin occurs, the temperature has a significant effect on which of the isomers is faster in the reaction. At room temperature, the *Z*-isomer is faster and more stable, whereas at high temperature and in a non-polar solvent, the *E*-isomer undergoes cycloaddition the fastest and most stable. The used reaction conditions could drive the nitrone isomerization equilibrium towards the *E*-nitrone conformation. We used nitrone (**1g**) as a model, where the methyl protons of the *Z*-isomer had chemical shifts in the 3.85 ppm range. On the other hand, peaks of the methyl protons for the E-isomer were assigned in the 3.90 ppm range. Also, the proton of CH = N of the E-isomer had chemical shifts at approximately 7.30 ppm, while that of the *Z*-isomer had chemical shifts of approximately 7.25 ppm. This is indicated by chemical shifts ∼0.05 ppm further upfield than the corresponding *Z*-isomer. The special nature of the olefinic bond substituted with an oxygen atom must play a significant role in the stereochemistry of these cycloaddition reactions. [3 + 2] Dipolar cycloaddition reactions of nitrones to olefines lead to the generation of two regioisomeric types of 4- or 5-substituted isoxazolidines. The electronic characteristics of the substituents on alkenes governed the regiochemistry. The isomer (5-substituted isoxazolidine) is produced predominantly by combining the nitrones with electron-rich alkenes, whereas in the instance of the olefine substituted with any electron-withdrawing group such as carbonyl or cyano, a reversal of regioselectivity is predominating as illustrated in Scheme 2. In alkenes that include electron-withdrawing groups, the interaction of the HOMOdipole with the LUMO dipolarophile is favored. This is attributed to the decrease of the double bond's orbital energy, which results in the creation of 4-substituted isoxazolidines. The olefin used in this study was (3,9-Divinyl-2,4,8,10-tetra oxaspiro (5-5) undecane), which can be considered as monosubstituted alkene and carries electron-donating substituents. This type of alkenes favors interaction between the LUMO dipole and the HOMO dipolarophile and, as expected, the 5-substituted isoxazolidine isomer is generated. The reaction was found to be extremely regioselective to the formation of only 5-substituted isoxazolidines. The electron-withdrawing effect of the benzene ring is the reason that the nitrones (**1a-l**) have an ionization capability more than normal nitrones. Thus, the interactions of the lowest unoccupied molecular orbital LUMOnitrone with the highest occupied molecular orbital HOMOalkenes have completely dominated the reaction and led to the production of solely 5-isoxazolidine adducts. The interaction takes place in polar reactions between the more electrophilic atoms of the alkene, the C5 carbon, and the more nucleophilic atoms of the nitrone, the oxygen atom.

**Scheme 3:**
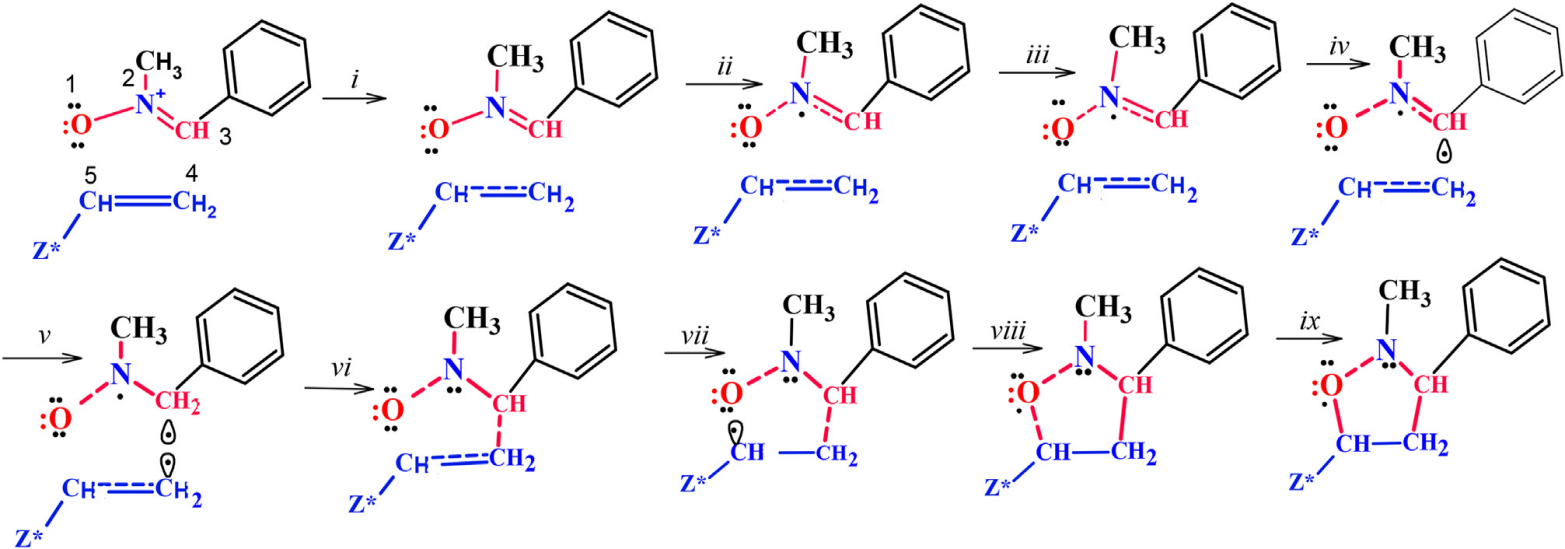
Exo Reaction route of the Nonpolar 1,3-dipolar cycloaddition reaction between Nitrone 1a and dipolarophile.

The yield of the products displays that the cycloaddition reaction occurred chemoselectivety furnishing bis-5-isoxazolidine). Mono-isoxazolidine might be formed and it was detected in some of the products by spectroscopic analysis. This reaction shown: (*I*) regioselective, as isomers **3** and **4** by addition the oxygen of the nitrone to the C5-carbon of the alkene. The reaction's mechanism is distinguished by ten differentiated phases [31] that can be classified into three groups (see Scheme 3): (a**) First group A**, which includes Phases ***i−iv***. This phase begins with the rupture of the double bonds of the reactants the N2–C3 and C4–C5, respectively, this leads to the formation of a lone pair of electrons on N2 on the N2 nitrogen in the scope of nitrone. (b) **Second group B**, which includes Phases ***v*** and ***vi*** which start with the creation of the two *pseudoradical* centers C3 (first), then C4 (second) at interactions between different carbons that are involved in the formation of the following C3–C4 as a new single bond. Ultimately, (c) **Third group C**, that includes Phases ***vii−x*** with the creation of the two new single bonds between C3 with C4 (first) and O1with C5 (second) and formation of either bis-5-isoxazolidines **4** or mono-5-isoxazolidines **3**. (*II*) stereoselective, the stereochemical relationship between the aryl ring and the C-5 moiety must be either cis or trans in the isoxazolidine rings but based on the ^1^H NMR spectra confirmed the structure of the cycloadducts. The analysis of products configuration indicated that the relationship between the aryl ring in isoxazolidine rings of most of the compounds **4a-l** and substituted carbon number C-5 are cis. On the background of the theoretical studies, the regioselectivity of Exo/Endo pathways in a 1,3-dipolar cycloaddition reaction is influenced by the electronic nature of the alkene, which is also affected by reactant size [32]. An endo approach occurs in polar reactions involving alkenes with electron-withdrawing substituents. Favorable electrostatic interactions between the two polarized reactants that are responsible for the endo stereoselectivity in polar reactions arises along with the endo approach.

**Scheme 4:**
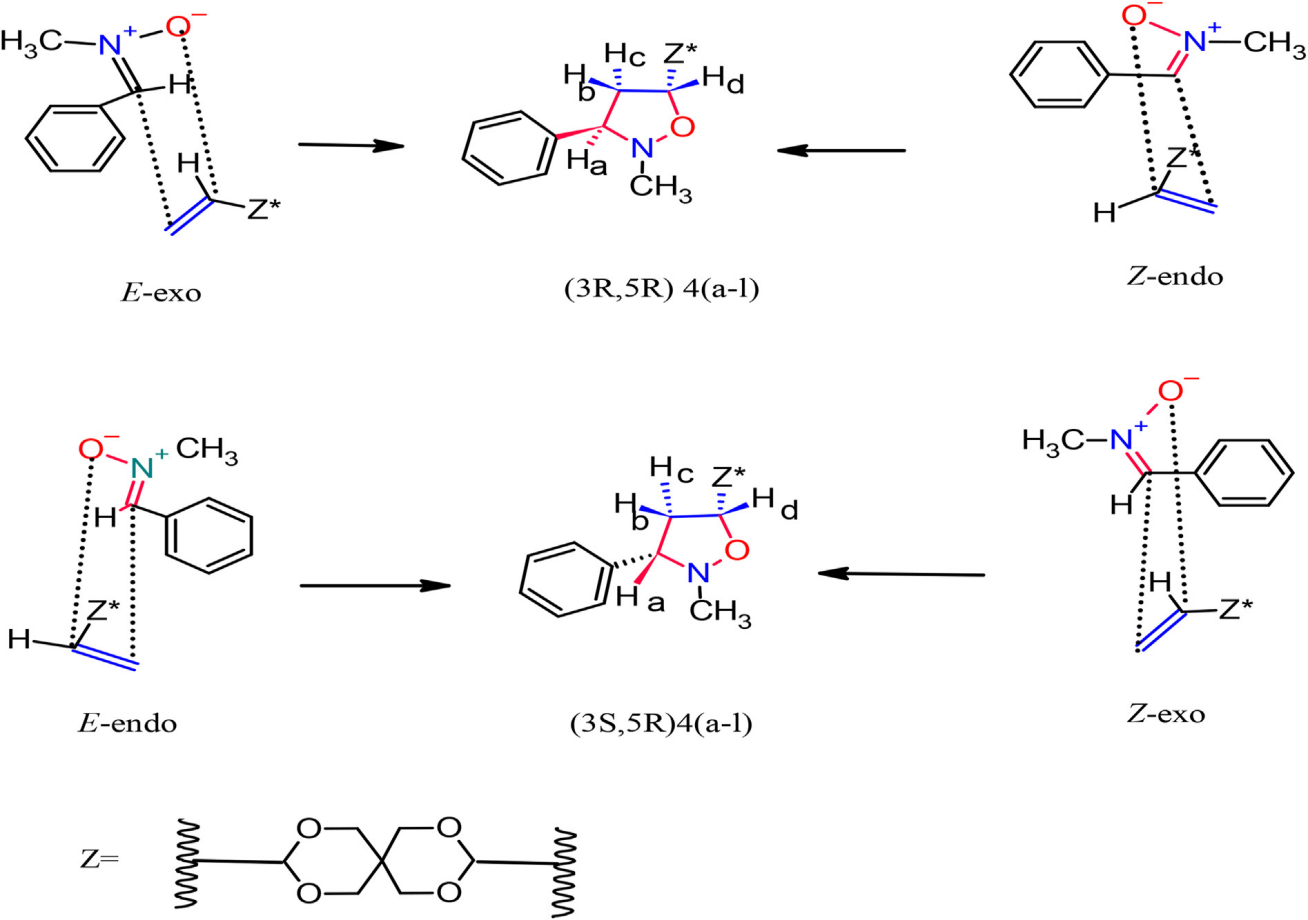
Four possible attacks to nitrone.

**Figure 1:**
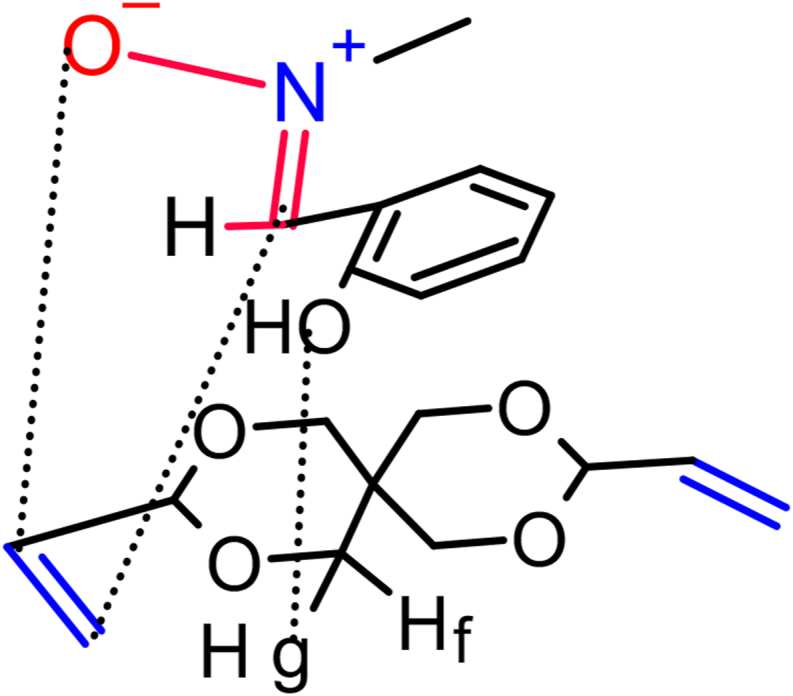
Electrostatic interactions between OH proton on aryl ring in nitrone with Hg proton in alkene by *E*-Endo-mode of isoxazolidine (4g).

**Figure 2:**
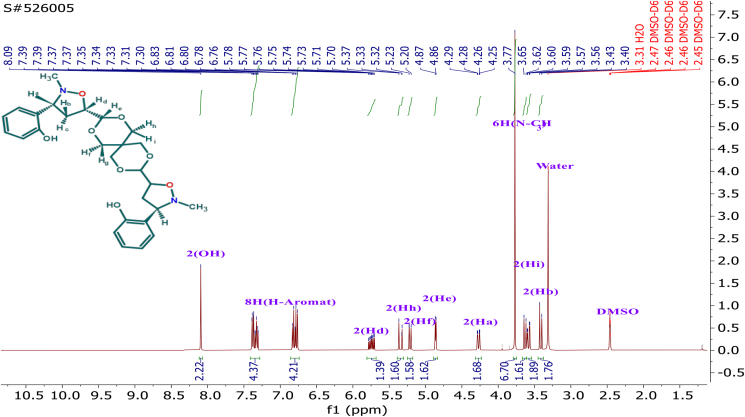
^1^H- NMR Spectrum for isoxazolidin (4g).

In nonpolar reactions that involve alkenes containing electron-releasing groups, due to the appearance of the unfavorable steric hindrance along the endo approach makes the *Exo* pathways more favorable, hence the bulky substituents are arranged in an *Exo* mode [31]. Liu et al. [33] performed DFT calculations on the 1,3-dipolar cycloaddition reactions of simple nitrones to dipolarophiles containing electron-donating-substituents. They found that the endo approach is kinetically favored due to stabilizing secondary orbital interactions, this is what we found in our research. Most of the results revealed that the attacking way was an *Endo* attack of *E*-nitrone. We also do not rule out the occurrence of the *Exo*-attack of *Z* nitrone, but its percentage is small according to the amount of the *Z* nitrone in the reaction, as usual in this type of cycloaddition as in Scheme 4. The regiochemistry of the cycloadducts was determined by standard spectroscopic studies. The ^1^H-NMR spectrum, of compound (**4g**) was symmetric as in Figure 2 showed two (ddd) at lower shift in the range δ = 3.60–3.57 ppm for Hb (ddd, *J*_b, c_ = 11.5, *J*_b, d_ = 8.2, *J*_b, a_ = 6.3 Hz) and δ = 3.43–3.40 ppm for Hc(ddd, *J*_c,b_ = 11.6, *J*_c,d_ = 5.6, *J*_c, a_ = 2.4 Hz) characteristic of the protons of the methylene in isoxazolidine ring. The presence of two (ddd) in ^1^H-NMR spectrum instead of two doublet of

doublet for diastereotopic methylene protons Hb, Hc in isoxazolidine ring, which are supposed to appear at a high chemical shift compared to a previous study literature [32], strongly excluded the formation of other regioisomers **5** (bis4,5- isoxazolidine) or **6** (bis-4-isoxazolidine). The isoxazolidine ring diastereotopic methylene protons are nonequivalent (have different chemical shifts). The five-membered ring prevents rotation, causing protons Hb and Hc to have different chemical shift values. That the Hb and Hc protons show (ddd) between δ = 3.60–3.40 ppm supports the formation of regioisomer **4g** (bis-5- isoxazolidine). H-a observed as a doublet of doublets at δ 4.27 (*J*_a, b_ = 2.4, *J*_a, c_ = 11.2 Hz) from their coupling constant values the relationship between H-a and H-b is cis and the relationship between H-a and H-c is trans. In cis adducts the relative configurations of H-b, H-a, and H-d protons of most of the cycloadducts are cis and most cycloadducts are in favor of E-endo or Z- Exo transition state geometry as obvious from their coupling constant values (*J*_Hb, Hd_ = 4.2 Hz). The absence of Hg proton in the H-NMR spectrum for the compound (**4g**) denoted those electrostatic interactions occurred between two reactants as in Figure 1.

**Figure 3:**
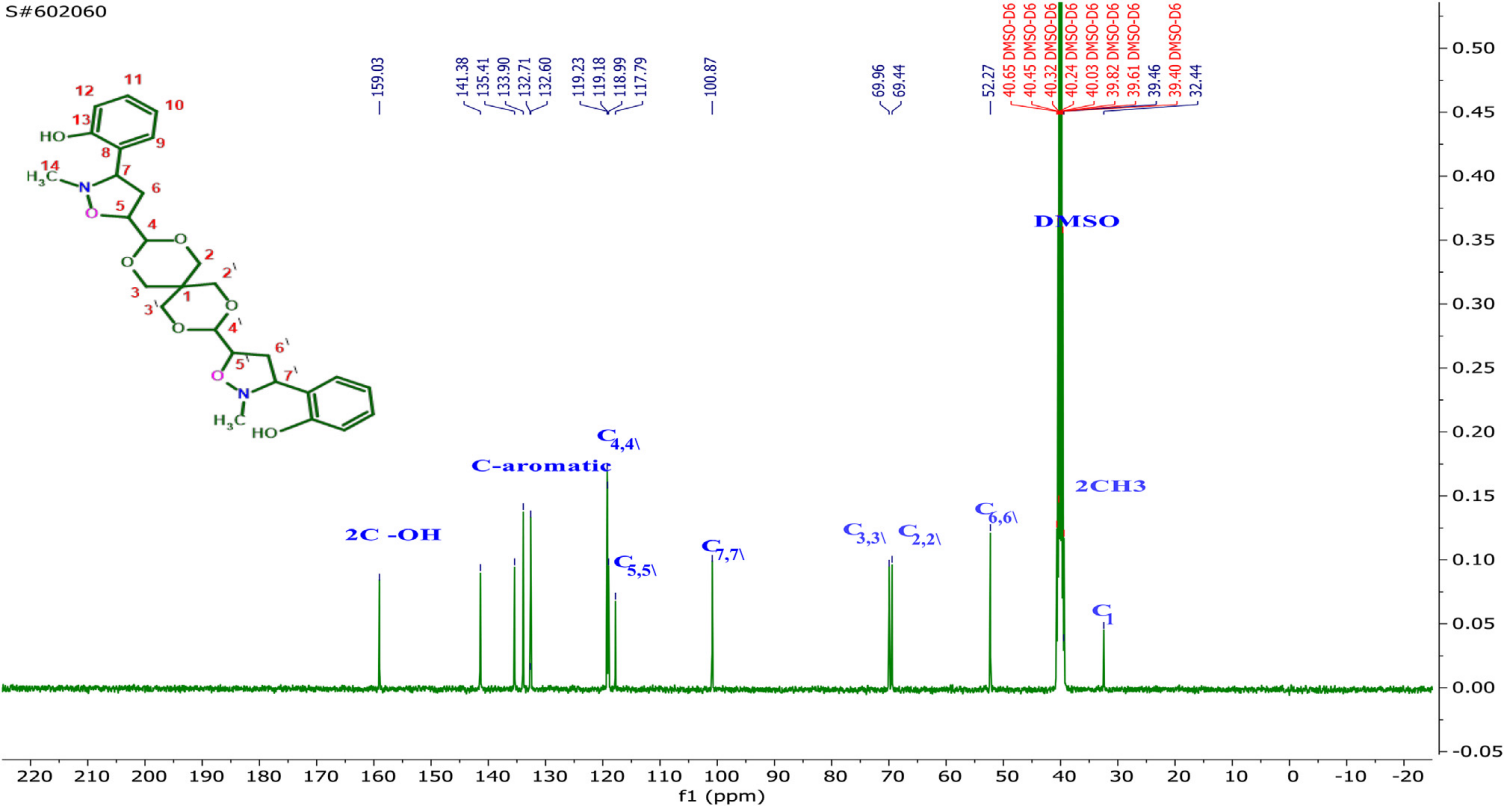
^13^C-NMR Spectrum for isoxazolidine (4g).

**Figure 4:**
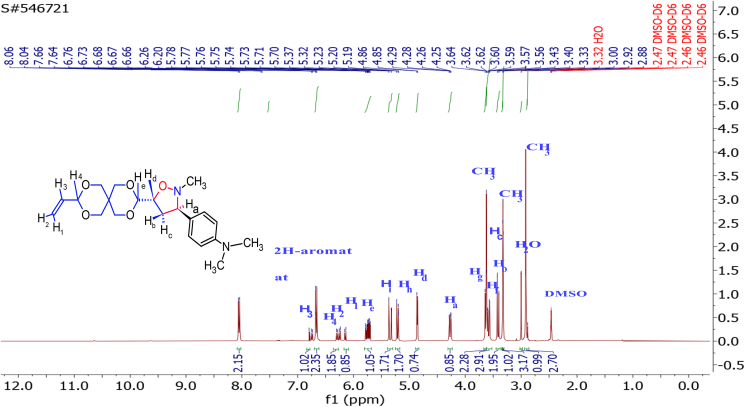
^1^H-NMR Spectrum for isoxazolidin (3b).

**Figure 5:**
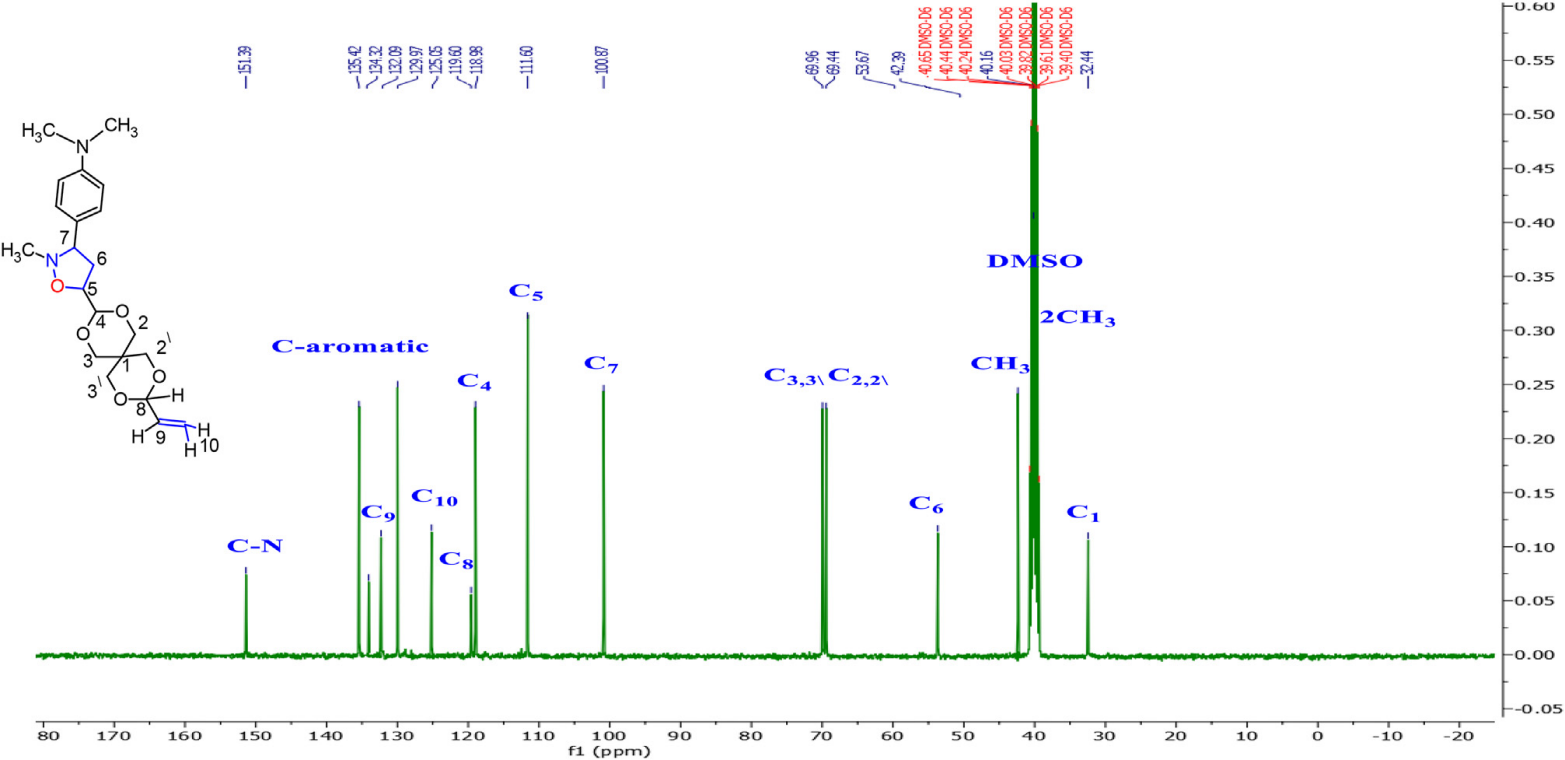
^13^C-NMR Spectrum for isoxazolidin (3b).

The interaction of the OH proton on the aryl ring in nitrone with the Hg proton for methylene protons of dipolarophile by the intermolecular hydrogen bonding leads to a reduction of the LUMO_dipolarophile_ energy level and a lowering of the energy gap between the LUMO_nitrone_/HOMO_alkene_ [32]. In the cycloaddition reaction during the formation of the transition state, the regioselectivity of adducts was also influenced by this interaction, so the resulting *endo*-mode (or *cis*) type geometry is more favorable in this case by *E*-nitrone. The ^13^C NMR spectrum of **4g** (Figure 3) exhibited peaks at δ = the signals at 117.79, 100.89 and 52.27 ppm characterize C5,5^∖^, C7,7^∖^, and C6,6∖ of the isoxazolidine ring, respectively. Through the detailed investigation of the nature of these cycloaddition reactions by ^1^H-NMR spectrum studies of the cycloadducts Figures S32-S49 (supplementary file), it was also confirmed that no diastereomers have been formed. As it is clear in Table 1 all compounds have undergone the cycloaddition reaction and gave the symmetry isomer **4** (bis-5-isoxazolidine), except of compounds **3b, 3e**, and **3k** that formed only mono-5-isoxazolidine because of the steric hindrance of bulky substitutes caused by the mono-5-isoxazolidine ring on the second cycloaddition reaction or via retro-cycloaddition leading to the format-ion of bis-isoxazolidines. As we mentioned previously, the compounds **3b, 3e**, and **3K**, through H-NMR spectroscopic analysis, were produced in the form of mono-5-isoxazolidine. The cycloaddition reaction took place on one side of the symmetric olefin and did not occur on the other side due to steric hindrance exerted by the bulky substituents on the benzene ring of the nitrone. We took the compound **3b** as a model. The protons of the isoxazolidine ring in Figure 4 appeared at chemical shifts in the range δ = 3.33ppm for Hb (dt *J*_c, b_ 11.6, 2.4 Hz) and δ 3.41 ppm for Hc (dt, J_b, c_,11.5, 8.3 Hz) corresponding to isoxazolidine ring methylene. The proton Ha is observed at 4.27ppm as a doublet of doublet (dd, *J*_a, b_11.3, J_a, c_ 2.4 Hz), while the peak at 5.74 ppm is assigned to Hd which appeared as a doublet of doublet of doublet (ddd, *J*_d, e_ 15.5, *J*_d, c_ 10.4, *J*_d, b_ 4.4Hz). The characteristic bands of the double bond protons H1, H2 appeared in the range of 6.20–6.26 ppm as a doublet. H4 is observed as a doublet overlapped with H2. In addition to the double bond protons, H3 has appeared at 6.78 ppm as a multiplet. H-NMR data clearly shows a *cis* relation between Ha and Hc protons, and between Hb and Hd protons, respectively, and this complies with previously reported data [34]. The trans adducts (3R, 5R) are produced by the *Exo* approach on the *E*-nitrone or the *Endo* approach on the *Z*-nitrone as depicted in Scheme 4. The ^13^C-NMR spectrum of **3b** exhibited peaks at 111.60, 100.87, and 53.67 ppm (Figure 5), which characterize C5,5^∖^, C7,7^∖^, and C6,6^∖^ of the isoxazolidine ring, respectively. On the other hand, the chemical shifts at δ 119.60, 125.05, and 132.09 ppm are for carbon atoms C8, C10, and C9, respectively. The highly symmetrical nature of the derivative **4c** was determined through the ^1^H-NMR spectrum. In the ^1^H-NMR spectrum, all the protons in the isoxazolidine ring Hb, Hc, and Hd appeared as multiples, except proton Ha, which was observed as two peaks isolated. This indicated that once Ha was cis and the other trans. Based on the spectral details, it was assured that the cycloaddition occurred at one of the double bonds via the E-endo approach and the other by the *E-exo* approach.

**Table 1:** Yield of isoxazolidines.

	Comp.	a	b	c	d	e	f	g	h	i	j	k	l
The isolated yield of cycloadducts (%)	3	-	76	-	-	79	-	-	-	-	-	78	-
	4	88	-	74	87	-	95	69	82	65	94	-	95
	5	-	-	-	-	-	-	-	-	-	-	-	-
	6	-	-	-	-	-	-	-	-	-	-	-	-

**Figure 6:**
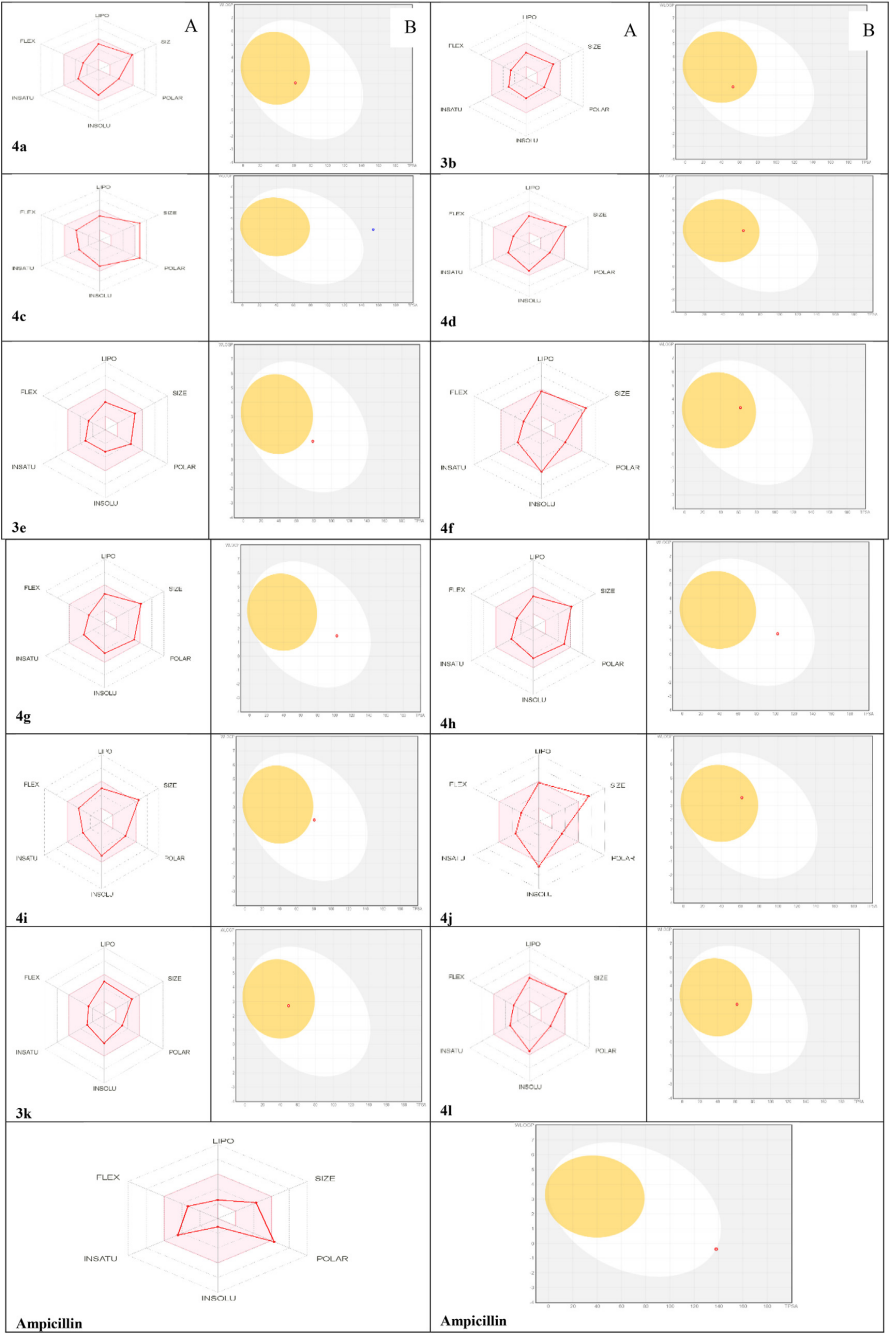
**(A)** Bioavailability radar; **(B)** the Boiled- Egg graph of compounds (3,4(a–l)) the white ellipse defining intestinal absorption, and the yellow ellipse defining brain permeation using Swiss ADME predictor.

The NMR spectral data were mentioned in the experimental part and spectra are in Fiures S34, S35 (supplementary file). Also, the trans product **4i** showed a highly symmetrical nature, as was ascertained by the ^1^H-NMR spectrum. The protons of the isoxazolidine ring were observed at chemical shifts in the range δ = 3.43 ppm for Hb (dt *J*_c, b_ 11.6, 2.4 Hz) and δ 3.63 ppm for Hc (dt, *J*_b, c_ 11.4, 4.3 Hz), corresponding to isoxazolidine ring's methylene. The proton Ha is observed at 4.24 ppm as a doublet of doublet (dd, *J*_a, c_11.3, *J*_a, b_ 2.4 Hz, while the peak at 5.73 ppm is assigned to Hd which appeared as a doublet of doublet of doublet (ddd, *J*_d, e_ 15.5, *J*_d, b_ 10.4, *J*_d, c_ 4.4Hz). We conclude from this that there is a cis relationship between Ha and Hc and between Hb and Hd protons, respectively. Therefore, the method of addition was by either the Exo mode on the (E)-nitrone or endo mode on the (Z)-nitrone.

## 4 Antimicrobial screening

Many compounds which have heterocyclic nuclei contained on N-bridge are described with antiviral and antimicrobial activity as isoxazolidines. According to the obtained MIC values found that all the novel synthesized analogs exhibited various degrees of antimicrobial activity against all the tested microorganisms when compared to the positive control. With some of the tested compounds demonstrated high activity and others being moderately active. The obtained data in Table 2 shows that compound (**4l**) had stronger antibacterial capabilities than the standard medicine, particularly (Ampicillin); it exhibited higher potency against *S.aureus* and *S.pyogenes* than Ampicillin, whereas it was equipotent to Ampicillin against *E.coli*. Also it showed weakly sensitivity against *P.aeruginosa* compared to Chloramphenicol drugs. On the other hand the compounds **3b,4c** revealed potency higher than Ampicillin against *S. aureus,* they showed approximate similarity to the effect of Ampicilli against *S.pyogenes.* Whereas compound (**3e**) displayed antibacterial activity little less than Ampicillin against *E. coli, S. pyogenes.* In contrast, compound (**3e**) was approximate similarity to the effect of Ampicillin against *S. aureus.* As illustrated in Table 2**.** Compound (**4g**) showed an impressive activity against *E. coli, S.pyogenus* higher than that of Ampicillin, and equal to that of Chloramphenicol. Similarly, it displayed potent activity against *S. aureus* similar to that of Ampicillin. In addition, the derivative (**4h**) showed equipotent activity to Chloramphenicol against *P. aeruginosa*. Compound (**4i**) displayed lower activity against *E. coli* and *S. pyogenes* compared to Ampicillin and it showed higher activity than Ampicillin against *S. aureus*. Compared to Chloramphenicol drug, all compounds showed modereate to weak activity, except the compound **4g** exhibited activity close of Chloramphenicol MIC value (62.5 μg/mL) against *E. coli*, showed equipotent activity to Chloramphenicol *S. pyrogens* MIC value (50 μg/mL), the compound **4h** exhibited approximate similarity to the effect of Chloramphenico *P. aeruginosa* MIC value (50 μg/mL), also the compound **4l** exhibited activity close of Chloramphenicol MIC value (62.5 μg/mL) *S.aureus*, showed equipotent activity to Chloramphenicol *S. pyrogens* MIC value (50 μg/mL), In addition to *S.aureus*, *E.coli*, *S.pyogenus,* and *P.aeruginosa* we used other kinds of fungus in our search. the fungus were *c.albicans*, *A.niger* and *A.clavatus*. The detailed results are shown in Table 2.

**Table 2:** MIC values (μg/mL) of some of the synthesized compounds.

ANTIBACTERIAL ACTIVITY					ANTIFUNGAL ACTIVITY		
minimum inhibition concentration					minimal fungicidal concentration		
Compound	*E. COLI*	*P. AERUGINOSA*	*S. AUREUS*	*S. PYOGENUS*	*C.ALBICANS*	*A. NIGER*	*A. CLAVATUS*
3b	125	100	**125**	**100**	**500**	250	250
4c	125	250	**125**	**100**	1000	500	500
3e	125	100	**250**	125	**500**	1000	1000
4g	**62.5**	100	**250**	**50**	1000	500	1000
4h	125	**50**	500	125	**500**	500	1000
4i	125	250	**125**	250	**250**	>1000	500
4l	**100**	250	**62.5**	**50**	**250**	500	>1000
Ampicillin	100	--	250	100	-	-	-
Chloramphenicol	50	50	50	50	-	-	-
Nystatin	-	-	-	-	100	100	100
Greseofulvin	-	-	-	-	500	100	100

**Table 3:** DPPH evaluate the antioxidant activity of studies compounds.

Entry	Concentrations (μg/mL)								
	1	2	5	10	25	50	100	200	IC _50_ μg/ml
3b	3.00	4.43	14.94	29.40	33.63	34.96	42.12	50.22	19.66 ± 0.68
4c	3.12	5.06	15.51	29.87	34.08	35.40	42.50	50.55	40.77 ± 1.41
3e	3.22	4.75	15.22	29.64	33.86	35.18	42.31	50.38	18.12 ± 0.63
4g	3.04	8.86	18.88	32.67	36.71	37.98	44.80	52.53	14.05 ± 0.49
4h	4.46	10.20	20.07	33.66	37.64	38.89	45.61	53.22	11.15 ± 0.39
4i	8.56	14.05	23.50	36.51	40.32	41.51	47.94	55.23	7.86 ± 0.27
4l	14.02	19.18	28.07	40.30	43.88	45.00	51.05	57.90	5.90 ± 0.20
Ascorbic acid									5.31 ± 0.18

The data are given in mean ± S.D of three independent experiments.

IC_50_ (μg/mL): values represent the quantity of extract required to scavenge 50% of the radicals contained in the reaction mixture.

The obtained data revealed that compound (**3b**) has high activity against *c. Albicans* similar to that Greseofulvin drug, moderated activity against another microorganism. Whereas The derivatives (**4c, 4g**) demonstrated weak activity against all fungus. Compounds (**3e, 4h**) showed equipotent activity to Greseofulvin drug against *c. albicans*, weak activity against another fungus. Finally derivative (**4i, 4l**) exhibited high activity against *c. albicans* more than Greseofulvin drug, moderated activity against other the tested strains. Compared to Nystatin drug, all compounds showed weak activity against the tested strains.

## 5 Antioxidant activity

The *in vitro* antioxidant capacity of samples (3b, 4c, 3e, 4g, 4h, 4i, 4l) was first determined by utilizing a synthetic reagent, 2,2 -diphenyl-1-picrylhydrazyl (DPPH), and was investigated according to the same technique as reported in reference [35]. For comparison, an ascorbic acid solution was utilized as a positive control. Table 3 shows the DPPH radical scavenging activity data indicated that all compounds have various inhibitory effects in a dose-dependent way upon both the absorbance of the DPPH according to the scavenging capabilities of studied compounds and IC_50_ values. The radical scavenging capacity of selected compounds increased in the following order: (**4c**) < (**3b**) < (**3e**) < (**4g**) < (**4h**) < (**4i**) < (**4l**) < Ascorbic acid. The IC_50_ value of a compound is the amount of the compound required to inhibit the DPPH free radical by 50%. The lower the IC_50_ value, the better the compound's capacity to inhibit. Additionally, the reported results exhibited that derivative (**4l**) possesses the highest antioxidant activity in the series with IC_50_ = 5.90 ± 0.20 μg/mL when compared to standard, ascorbic acid (IC_50_ = 5.31 ± 0.18 μg/mL). while the lowest one was the compound (**4c**) IC_50_ = 40.77 ± 1.41 μg/mL. The antioxidant activity was calculated in terms of Radical Scavenging Activity (RSA) by Eq. (1).

(1) RSA = *A*DPPH−*A* ×100%*/A*DPPH

The RSA (%) for isoxazolidine derivatives (**3b, 4c, 3e, 4g, 4h, 4i**, **4l**) has been illustrated in Table 2 at eight different concentrations (1, 2, 5, 10, 25, 50, 100, and 200 μg/mL) of the selected compounds with DPPH at 517nm. Figure 4 depicts the percentage of antioxidant activity at different concentrations and IC50. In an attempt to understand the effect of different functional groups on the antioxidant behavior of novel isoxazolidine derivatives **3b, 4c, 3e, 4g, 4h, 4i, 4l** and to gain knowledge of some structure-activity relations established on the existence of different substituents on the phenyl ring, The electronic characteristics of the phenyl ring substituents have a significant effect on their antioxidant effectiveness, according to chemical structural characteristics. According to our findings, the antioxidant capability of the tested derivatives is directly affected by the substituents on the phenyl ring. The antioxidant behavior of the selected compounds has followed this sequence: 4-CH_3_> 4–OCH_3_> 4–OH> 2-OH >4-OH-3-OCH_3_>4-N-(CH_3_)_2_ >2-N_2_O. Further investigation of tested synthesized isoxazolidines revealed that the presence of methyl, methoxy, amine, and hydroxyl groups at the para position in the aromatic ring, having high electron-donating properties, activated the aromatic ring and increased the scavenging activity.

## 6 Physicochemical properties

To support the congruence of in vitro biological potency and drug-likeness, an in silico ADME study was carried out using the SwissADME predictor, which is studied as a crucial part that gives the basis for molecules to be effective oral drug candidates. To predict whether a compound is a potential drug candidate, various rules are used like the Lipinski's rule of five: molecular weight 500 Da, hydrogen bond acceptors <10, H-bond donors <5, TPSA< 140 Å^2^, and a lipophilicity value of Log P < 5. If two or more violations were found, the candidate compounds were deemed unacceptable for the drug-likeness [36, 37]. Accordingly, all derivatives displaying drug-likeness features follow Lipinski's rule of five except the compound **4c**. The synthesized compounds have a number of rotatable bonds in the range of 4 ≤ Nrotb ≤6, indicating their low structural flexibility, which makes them potentially bioavailable orally. A number of hydrogen bond acceptors (6 ≤ NON ≤12) and hydrogen bond donors (0 ≤ NOHNH ≤2) follow Lipinski's rule of five and, as a result, enhance their oral drug-like qualities (except compound **3c**). The outcomes in Table 4 exhibited that compound **4c** had two violations and a bioavailability score of 0.17. Most of the compounds had a bioavailability score of about 0.55. TPSA values for compounds were in the range of 153.50 to 49.39 Å^2^, most of the compounds having TPSA values of 82.32 Å^2^, implying high intestinal absorption and BBB penetration, making compounds **4a, 3b, 4d, 3e, 4f, 4j, 3k, and 4l** suitable pharmacological candidates for further development. The % ABS of most derivatives was high (>80 %), indicating good oral absorption. Typically, compounds with greater than 80% absorption have good oral absorption and can be regarded as good drugs. All compounds gave XLogP values of less than five, indicating good permeability and, as a result, good lipid solubility, which will allow the drug to cross the cell membrane and be used to generate bioactivity. The pharmacokinetic properties as shown in Table 4 indicate that all the derivatives are predicted to possess good GI absorption. In addition, they were predicted to be not P-gp substrates, providing their favorable intestinal absorption and bioavailability, and most of the compounds (**4a, 3b**, **4d, 3e, 4f, 4j, 3k, 4l**) can pass the blood-brain-barrier (BBB), indicating their ability to penetrate the BBB and exert action in the central nervous system. The pharmacokinetics was evaluated by the Boiled- Egg model as illustrated in Figure 6. If the compounds are in the white ellipse, they are expected to be well absorbed by the gastrointestinal tract. Whereas the yolk (yellow ellipse) means that the contained compound can pass the blood-brain-barrier (BBB). Compounds **4a, 3b, 4d, 4f, 4j, 3k,** and **4l** exhibited high brain penetrant, as demonstrated by their location inside the yellow ellipse, while compounds **3e, 4g, 4h**, and **4i** were predicted to have a high intestinal absorption, as demonstrated by their location inside the white ellipse. As a result, they do not affect drug excretion and don't have any inhibitory effects on the five CYP enzymes: CYP1A2, CYP2D6, CYP2C19, CYP3A4, and CYP2C9, which suggests that they don't have any toxic ADME qualities without buildup of the drug. Their negative skin permeability values of -8.04 ≤ log ≤ -6.43 demonstrate that there is little skin penetration. The physicochemical properties suggest that the number of violations of Lipinski's rule of five is 0–1 as indicated in Table 4. Oral absorption ranges from 80.4% to 92% for many of the compounds. In other words, practically all the compounds' characteristics are within the acceptable range, and this means that all of the examined molecules are likely to be useful as therapeutic candidates. These compounds' medicinal chemistry features were anticipated, and the findings demonstrated that synthetic accessibility ranged from 5.24 to 6.20, indicating that all of them have a good possibility to be synthesized. Bioavailability radars may be used to easily determine drug-likeness features of the tested molecules, which are represented in the pink region by the optimal scope for each feature (lipo, size, solubility, polarity, flexibility, and saturation). As Figure 6 reveals, all designed isoxazolidines, their polygon of physicochemical space for all parameters are within the ideal region, which means that all compounds have superior oral bioavailability. Because of their specified molecular properties, these compounds can be used as drugs orally.

**Table 4:** Physicochemical-Pharmacokinetic/ADME properties and drug-likeness predictions and of tested compounds. Here, MW = Molecular Weight, HBA = Hydrogen Bond Acceptors (O and N atoms <10), RB = Rotatable Bond <10, TPSA = Total Polar Surface Area, HBD = Hydrogen Bond Donors (OH and NH group <5), GIA = GI Absorption, Topological polar surface area. c %ABS = 109 – (0.3345 × TPSA), BBBP = Blood Brain Barrier Permeation, PgPS = P-Glycoprotein Substrate, LV = Lipinski Violation, LLV = Lead likeness Violations, BS = Bioavailability Score, SA = Synthetic Accessibility, S = Ampicillin.

Entry	4a	3b	4c	4d	3e	4f	4g	4h	4i	4j	3k	4l	S
Pharmacokinetics													
MW (< 500 Da)	482.57 g/mol	390.47 g/mol	572.56 g/mol	518.55 g/mol	393.43 g/mol	551.46 g/mol	514.57 g/mol	514.57 g/mol	542.62 g/mol	640.36 g/mol	389.48 g/mol	510.62 g/mol	349.40 g/mol
RP	4	4	6	4	4	4	4	4	6	4	4	4	5
HBA (< 10)	8	6	12	10	8	8	10	10	10	8	6	8	5
HBD (< 5)	0	0	0	0	1	0	2	2	0	0	0	0	3
TPSA	61.86 A^2^	52.63A2	153.50 A^2^	61.86 A^2^	78.85A^2^	61.86 A^2^	102.32A^2^	102.32A^2^	80.32 A^2^	61.86 A^2^	49.39 A^2^	61.86 A^2^	138.03 Å^2^
ABS%	88.30	91.39	57.65	88.30	82.62	88.30	74.77	74.77	82.13	88.30	92.47	88.30	62.82
Fraction Csp3	0.56	0.62	0.56	0.56	0.60	0.56	0.56	0.56	0.59	0.56	0.64	0.59	0.44
XLOGP3 (LogP < 5)	3.24	2.17	2.90	3.45	1.66	4.50	2.54	2.54	3.19	4.63	3.17	3.98	-1.13
GIA	High	High	Low	High	High	High	High	High	High	High	High	High	Low
BBBP	Yes	Yes	No	Yes	No	Yes	No	No	No	Yes	Yes	Yes	No
PgPS	No	No	Yes	No	No	No	No	No	No	No	No	No	No
CYP1A2 INHIBITION	No	No	No	No	No	No	No	No	No	No	No	No	No
CYP2C19 INHIBITION	No	No	No	No	No	No	No	No	No	No	No	No	No
CYP2C9 INHIBITION	No	No	Yes	No	No	No	No	No	No	No	No	No	No
CYP2D6 INHIBITION	Yes	Yes	No	Yes	Yes	No	Yes	Yes	Yes	No	Yes	No	No
CYP3A4 INHIBITION	No	No	No	No	No	No	No	No	No	No	No	Yes	No
Log Kp (cm/s)	-6.94	-7.14	-8.04	-7.01	-7.52	-6.47	-7.64	-7.64	-7.35	-6.92	-6.43	-6.59	-9.23
LV	0	0	2	1	0	1	1	1	1	1	0	1	0
BS	0.55	0.55	0.17	0.55	0.55	0.55	0.55	o.55	0.55	0.55	0.55	0.55	0.55
LLV	1	1	1	1	1	2	1	1	1	1	1	2	No
SA	5.97	5.26	6.20	5.99	5.24	5.32	6.06	5.98	6.18	6.02	5.39	6.18	4.16

## 7 Docking studies

**Figure 7:**
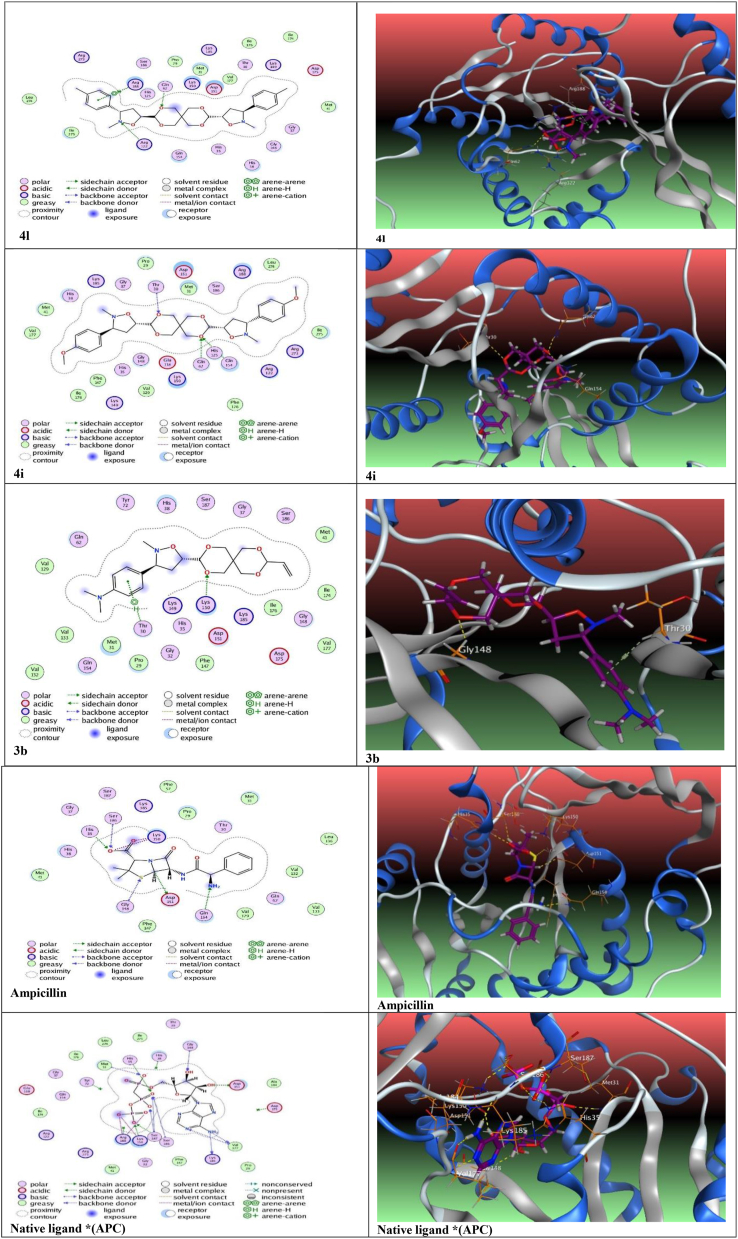
Interaction of selected compounds 4l, 4i, 3b, ampicillin, and Native ligand with 2 × 3F, both 2D (**left**) and 3D (**right**) diagrams, is given below with yellow dotted line in the 3D figure represents H-bond.

Through the results of the in vitro antimicrobial activity that we obtained for the synthesized compounds, it was found that in comparison to standard medications such as Ampicillin, some of the studied compounds (**3b, 4c, 3e, 4g, 4h, 4i**, **4l**) exhibit good biological activity against certain tested strains, even some of them are higher activity than the standard drug itself or it was equipotent to it. The measured activity varies from compound to compound, which could result in the basic skeleton's structural geometry and the presence of different substituted groups and heteroatoms. The results of docking studies including docking scores, hydrogen bonding interactions between amino acids residues, and functional groups of docked compounds, and their RMSD (the root-mean-square deviation of atomic positions must be less than 1.9 Å**)** were summarized in Table 5. When compared to Ampicillin, and native ligand, all the studied compounds had excellent binding energy values. All the compounds exhibited docking score values higher from standard drugs except the compound **4h** as shown in Table 5. The designed cycloadducts are docked towards the crystal structure of Methicillin-Resistant *Staphylococcus aureus* Sar2676, a Pantothenate Synthetase 2 × 3F to ascertain their inhibition activity as in Figure S50 (supplementary file). For docking studies with (2 × 3F), we selected the compounds (**4l, 4i, and 3b)** because they have the strongest inhibitor activity among all derivatives. The binding of the molecules to the active site of the protein is given in Figure 7. The range of docking scores is -5.71 kcal/mol (**4h**) to -8.32 kcal/mol (**4l**) when compared to the standard agent Ampicillin and, the native ligand found that the native ligand has a binding affinity docking score of (−8.69) kcal/mol with three hydrogen bonds, while ampicillin has a binding affinity docking score of (−7.01) kcal/mol with three hydrogen bonds too. The order of the synthesized compounds according docking score values from highest to lowest was as follows: **native ligand > 4l > 4i > 3b > 4h.>4g > 4c > 3e> Ampicillin >4h**. This arrangement is consistent with the compounds' activity against bacteria *S. aureus in vitro* especially the first three compounds as in Table 2. The best-docked compound is (**4l**) with -8.32 kcal/mol. It had a greater docking score than Ampicillin, Chloramphenicol, and close of the native ligand as shown in Table 5. The compound (**4l**) has a binding docking score of (−8.32) kcal/mol and exhibited stronger binding to the active site with four bonds more tightly than other compounds, where the compound formed two H- bonds with the amino acids Gln62 with a distance 2.93Å, Arg122 with length 3.31 Å. Additionally, compound **4l** formed one pi-H bond with Arg188 residue and forms only one pi-cation stacking with the same amino acid Arg188residue. On the other hand, the compound (**4i**) showed three H-bond possible interactions with amino acid Gln154 (H-acceptor) with distance 2.98A, and formed a second binding in active site with amino acid Gln62 (H-acceptor) with a distance 2.69 Å, and the third interaction was with the amino acid Thr30 (H-acceptor) with length 3.29 Å. It has a docking score of -8.21 kcal/mol. The compound 3b interacted with 2 × 3F and formed two bonds, one important H-acceptor interactions between the oxygen atom of dipolarophile moiety and H of Lys150 with abond length (2.88 Å) residue. The second interaction was pi-H bonding to the phenyl ring and H of amino acid Thr30 of length (2.82 Å) as depicted in Figure 7. The above results are in good agreement with the given in vitro inhibition activity and corroborate with our previous work.

**Table 5:** Docking studies for compounds 3b,4c,3e,4g,4h,4i,4l with PDB ID: 2X3F.

Comp.	Docking score	RMSD (Kcal/mol)	Residue	No. of interactions
3b	-8.20	1.23	LYS150, THR30	1 H-acceptor 1 pi- H
4c	-7.5	0.97	GLU118, LYS150, SER186, LYS150, ARG273GLN154	One H-donor, 4 H-acceptor
3e	-7.31	1.02	MET31, MET31, SER187	2 H-acceptor 1 pi- H
4g	-7.42	1.54	GLU118, ARG188	1 H-donor, 1 H-acceptor
4h	-5.71	1.79	HIS38, TYR72, SER187	3 H-acceptor
4i	8.21	1.61	GLN62, GLN154, THR30	3 H-acceptor
4l	-8.32	1.39	GLN62, ARG122, ARG188, ARG188	2 H-acceptor 1 pi- H 1 pi-cation
Ampicillin	-7.01	1.92	ASP151, GLN154, HIS35, SER186, GLY148, LYS150, LYS150	One H-donor, 4 H-acceptor 2 ionic
Chloramph -enicol	-5.94	1.6	GLN148, ARG188, LYS150	3 H-acceptor
Native ligand (APC)	-8.69	1.75	ASP151, VAL127, LYS185, LYS150, SER186, HIS35, SER186, SER187, SER187, MET31, GLY148, VAL177, ARG188, LYS150, LYS150, ARG188,	3 H-donor, 9 H-acceptor 3 ionic

∗Native ligand: DIPHOSPHOMETHYLPHOSPHONIC ACID ADENOSYL ESTER.

## 8 Conclusion

In conclusion, through 1,3-dipolar cycloaddition, we successfully designed and produced novel isoxazolidine compounds from easily accessible chiral C-aryl-N-phenylnitrones and 3,9-Divinyl-2,4,8,10-tetra oxaspiro (5-5) undecane as new categories of antioxidant and antibacterial agents. The cycloaddition proceeds regio- and stereoselectively generating mono-5-isoxazolidines and bis-5- isoxazolidines, as a predominant component, where the oxygen of the nitrone is linked to the 5-carbon in the alkene. The steric hindrance of bulky groups such as 4-N-dimethylamine, 4-isopropyl, and 3-methoxy-4-hydroxy in the phenyl ring of nitrone lead to the formation of solely mono-5-isoxazolidines. The cycloaddition of mono-5-isoxazolidines to bis-5-isoxazolidines exhibits facial diastereoselectivity that can be attributed to steric control. The biological activity study showed Compound **4l** has the highest bioactivity as the best antibacterial and antioxidant potential as compared to others. Compound **4l** can therefore be considered a hit for further investigations applications in medicinal chemistry. The physicochemical and ADME characteristics indicate that the title compounds have good oral bioavailability and, as a result, could be some compounds that showed good activity are promising hit candidates for further drug discovery of new antibacterial and antioxidant agents after a systematic in vivo examination. The synthesized derivatives were studied for their interactions with **2 × 3F** by a molecular docking protocol. The selected compounds demonstrated a good docking score values, and the results were agreement with the in vitro antibacterial assay. We studied the applications of these compounds as antimicrobials and antioxidants only, and the applications of isoxazolidins are many, so it is possible to study in the future these compounds as an anti-inflammatory, anticancer, and antidiabetes. Where a study predicting their candidacy as oral drugs were good.

## Declarations

### Author contribution statement

Arwa AL-Adhreai: Conceived and designed the experiments; Performed the experiments; Analyzed and interpreted the data; Wrote the paper.

Mohammed ALSaeedy: Analyzed and interpreted the data; Wrote the paper.

Ali Alrabie: Performed the experiments; Analyzed and interpreted the data; Wrote the paper.

Inas Al-Qadsy, ZabnAllah M. Alaizeri, Hisham A. Alhadlaq, Abdulrahman Al-Kubati, Maqusood Ahamed: Contributed reagents, materials, analysis tools or data.

Sam Dawbaa: Analyzed and interpreted the data.

Mazahar Farooqui: Conceived and designed the experiments; Analyzed and interpreted the data; Wrote the paper.

### Funding statement

This work was supported by King Saud University, Riyadh, Saudi Arabia (project number: RSP-2021/129).

### Data availability statement

Data will be made available on request.

### Declaration of interests statement

The authors declare no conflict of interest.

### Additional information

No additional information is available for this paper.

## Appendix A Supplementary data

supplementary file Heliyon supplementary file Heliyon
