# Supplementary material for: Design and synthesis of novel enantiopure Bis(5-Isoxazolidine) derivatives: insights into their antioxidant and antimicrobial potential via in silico drug-likeness, pharmacokinetic, medicinal chemistry properties, and molecular docking studies
Source: Heliyon. 2022 Jun 24;8(6):e09746. doi: 10.1016/j.heliyon.2022.e09746 (PMC9253851; doi:10.1016/j.heliyon.2022.e09746)
Supplement: supplementary file Heliyon [file mmc1.docx]

Design and Synthesis of Novel Enantiopure Bis(5-Isoxazolidine) Derivatives: Insights into Their Antioxidant and Antimicrobial Potential via In Silico Drug-Likeness, Pharmacokinetic, Medicinal Chemistry Properties, And Molecular Docking Studies

**Supplementary File**

Arwa AL Adhreai ^a^
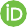
^**^, Mohammed ALsaeedy ^b^
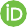
, Ali  [Alrabie](https://sciprofiles.com/profile/author/V3NjNER3NkRLMXlhS1ltOWJlUFY2bHRrYWRiTExURHJhdHVrbmxHVy9ncz0=) ^a^
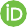
, Inas Al-Qadsy ^a^, Sam Dawbaa ^c^, ZabnAllah M. Alaizeri ^d^, Hisham A. Alhadlaq ^d^, Abdulrahman Al-Kubati ^e^, Maqusood Ahamed ^d^, Mazahar Farooqui ^a^
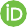
^*^

***^a^ Department of Chemistry, Maulana Azad of Arts, Science and Commerce, Aurangabad,*** ***431004****,* ***India***

***^b^ Department of chemistry, Faculty of Science, Anadolu University, Eskişehir, Türkiye***

***^c^ Department of Pharmaceutical Chemistry, Faculty of Pharmacy, Anadolu University, 26470, Eskişehir, Turkey***

***^d^ Department of Physics and Astronomy, College of Science, King Saud University, Riyadh-11451, Saudi Arabia***

***^e^ Department of Chemistry "Giacomo Ciamician" University of Bologna, 40126 Bologna BO, Italy***

**IR for Nitrones**

**Figure S1: IR for C-( phenyl)-N-methylnitrone (1a).**

**Figure S2: IR of C-(4- N-Dimethylphenyl)-N-methylnitrone (1b)**

**Figure S3: IR of C-(2- nitrophenyl)-N-methylnitrone (1c).**

**Figure S4: IR of C-(4- fluorophenyl)-N-methylnitrone (1d).**

**Figure S5: IR of C-(4- hydroxy-3-methoxyphenyl)-N-methylnitrone (1e)**

**Figure S6: IR of C-(4-chlorophenyl)-N-methylnitrone (1f)**

**Figure S7: IR of C-(2- hydroxyphenyl)-N-methylnitrone (1g).**

**Figure S8: IR of C-(4-methoxyphenyl)-N-methylnitrone (1i).**

**Figure S9: IR of C-(4-bromophenyl)-N-methylnitrone (1j).**

**Figure S10: IR of C-(4- methylphenyl)-N-methylnitrone (1l)**

**IR for isoxazolidines**

**Figure S11: IR of 2-methyl-5-(9-(2-methyl-3-phenylisoxazolidin-4-yl)-1,5,7,11-tetraoxaspiro [5.5] undecan-3-yl)-3-phenylisoxazolidine (4a)**

**Figure S12: IR of 4-(5-(9-(3-(4-(dimethylamino) phenyl)-2-methylisoxazolidin-4-yl)-1,5,7,11-tetraoxaspiro [5.5] undecan-3-yl)-2-methylisoxazolidin-3-yl)-N, N-dimethylaniline (3b)**

**Figure S13: IR of 2-methyl-5-(9-(2-methyl-3-(2-nitrophenyl) isoxazolidin-4-yl)-1,5,7,11-tetraoxaspiro [5.5] undecane-3-yl)-3-(2-nitrophenyl) isoxazolidine (4c)**

**Figure S14: IR of 3-(4-fluorophenyl)-5-(9-(3-(4-fluorophenyl)-2-methylisoxazolidin-4-yl)-1,5,7,11-tetraoxaspiro [5.5] undecan-3-yl)-2-methylisoxazolidine (4d)**

**Figure S15: IR of 4-(5-(9-(3-(4-hydroxy-3-methoxyphenyl)-2-methylisoxazolidin-4-yl)-1,5, 7, 11-tetraoxaspiro [5.5] undecan-3-yl)-2-methylisoxazolidin-3-yl)-2-methoxyphenol (3e)**

**Figure S16: IR of 3-(4-chlorophenyl)-5-(9-(3-(4-chlorophenyl)-2-methylisoxazolidin-4-yl)-1,5,7,11-tetraoxaspiro [5.5] undecan-3-yl)-2-methylisoxazolidine (4f)**

**Figure S17: IR of 2-(5-(9-(3-(2-hydroxyphenyl)-2-methylisoxazolidin-4-yl)-1,5,7,11-tetraoxaspiro [5.5] undecan-3-yl)-2-methylisoxazolidin-3-yl) phenol (4g)**

**Figure S18: IR of 3-(4-methoxyphenyl)-5-(9-(3-(4-methoxyphenyl)-2-methylisoxazolidin-4-yl)-1,5,7,11-tetraoxaspiro [5.5] undecan-3-yl)-2-methylisoxazolidine (4i)**

**Figure S19: IR of 3-(4-bromophenyl)-5-(9-(3-(4-bromophenyl)-2-methylisoxazolidin-4-yl)-1,5,7,11-tetraoxaspiro [5.5] undecan-3-yl)-2-methylisoxazolidine (4j)**

**Figure S20: IR of 3-(4-isopropylphenyl)-5-(9-(3-(4-isopropylphenyl)-2-methylisoxazolidin-4-yl)-1,5,7,11-tetraoxaspiro [5.5] undecan-3-yl)-2-methylisoxazolidine (3k)**

**Figure S21: IR of 2-methyl-5-(9-(2-methyl-3-(p-tolyl) isoxazolidin-4-yl)-1,5,7,11-tetraoxaspiro [5.5] undecan-3-yl)-3-(p-tolyl) isoxazolidine (4l)**

**^1^H-NMR and ^13^C-NMR for nitrones**

**Figure S22: ^1^H-NMR of** **C-( phenyl)-N-methylnitrone (1a).**

**Figure S23: ^13^C-NMR of C-( phenyl)-N-methylnitrone (1a).**

**Figure S24: ^1^H-NMR spectrum for C-(4-Floro)-N-methylnitrone (1d)**

**Figure S25: ^13^ C-NMR spectrum for C-(4-Floro)-N-methylnitrone (1d)**

**Figure S26: ^1^H-NMR spectrum for C-(4-Chlorophenyl)-N-methylnitrone (1f)**

**Figure S27: ^13^C-NMR spectrum for C-(4-Chlorophenyl)-N-methylnitrone (1f)**

**Figure S28: ^1^H-NMR spectrum for C-(4-Hydroxy)-N-methylnitrone (1h)**

**Figure S29: ^13^C-NMR spectrum for C-(4-Hydroxy)-N-methylnitrone (1h)**

**Figure S30: ^1^H-NMR spectrum for C-(4-methyl)-N-methylnitrone (1l)**

**Figure S31: ^13^C-NMR spectrum for C-(4-methyl)-N-methylnitrone (1l)**

**Fig. 31: ^13^ C-NMR spectrum for C-(4-Floro)-N-methylnitrone (1d)**

**^1^H and ^13^C-NMR for isoxazolidines**

**Figure S32: ^1^H-NMR for isoxazolidine (3b)**

**Figure S33: ^13^C-NMR for isoxazolidine (3b)**

**Figure S34: ^1^H-NMR for isoxazolidine (4c)**

**Figure S35: ^13^C-NMR for isoxazolidine (4c)**

**Figure S36: ^1^H-NMR for isoxazolidine (4d)**

**Figure S37: ^13^C-NMR for isoxazolidine (4d)**

**Figure S38: ^1^H-NMR for isoxazolidine (3e)**

**Figure S39: ^13^C-NMR for isoxazolidine (3e)**

**Figure S40: ^1^H-NMR for isoxazolidine. (4f)**

**Figure S41: ^13^C-NMR for isoxazolidine. (4f)**

**Figure S42: ^1^H-NMR for isoxazolidine (4g)**

**Figure S43: ^13^C-NMR for isoxazolidine(4g)**

**Figure S44: ^1^H-NMR for isoxazolidine (4h)**

**Figure S45: ^13^C-NMR for isoxazolidine (4h)**

**Figure S46: ^1^H-NMR for isoxazolidine (4i)**

**Figure S47: ^13^C-NMR for isoxazolidine(4i)**

**Figure S49: ^13^C-NMR for isoxazolidine(4l)**

**Figure S48: ^1^H-NMR for isoxazolidine (4l)**

**Figure 7 in the manuscript**

| 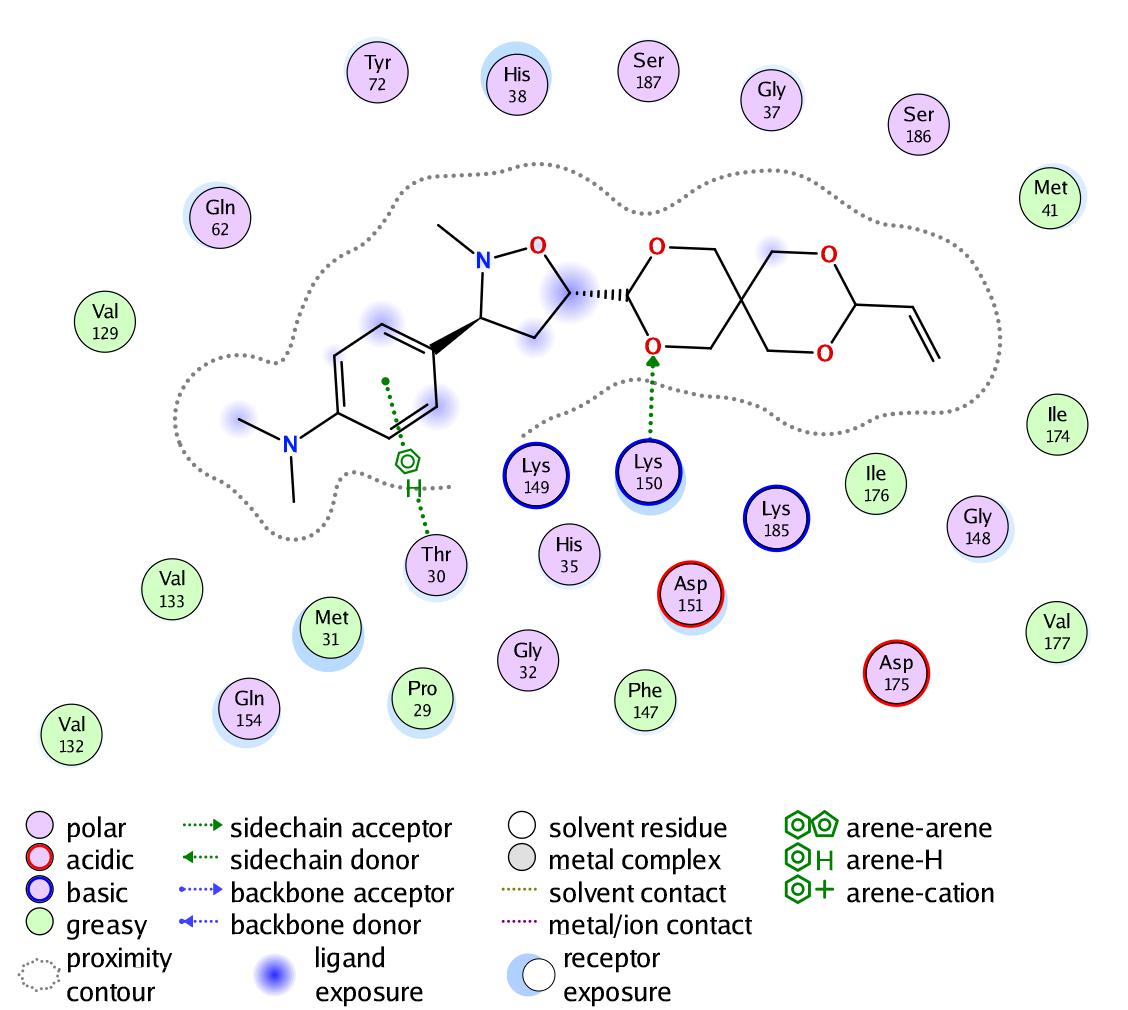  **3b** | 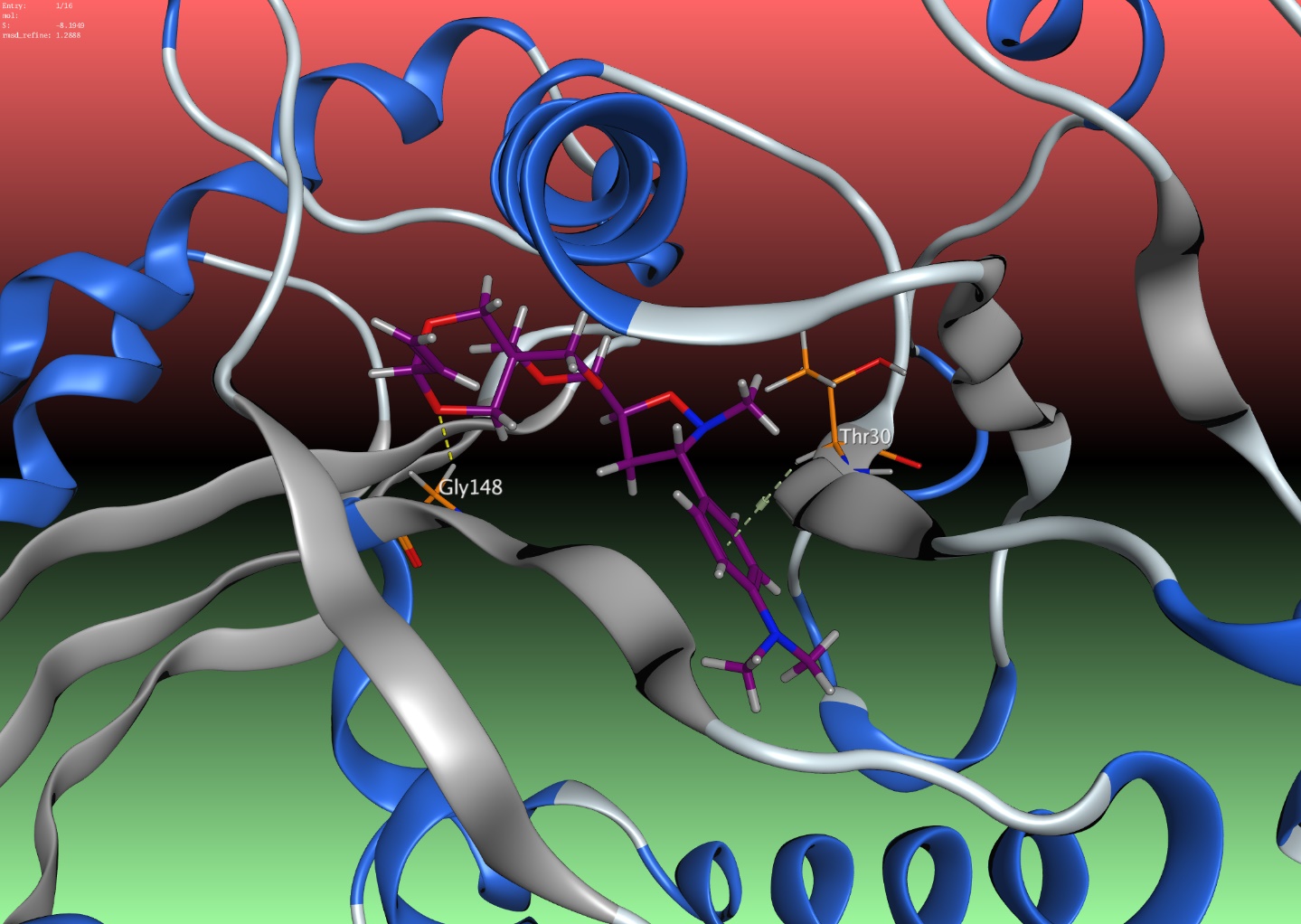  **3b** |
| --- | --- |
| **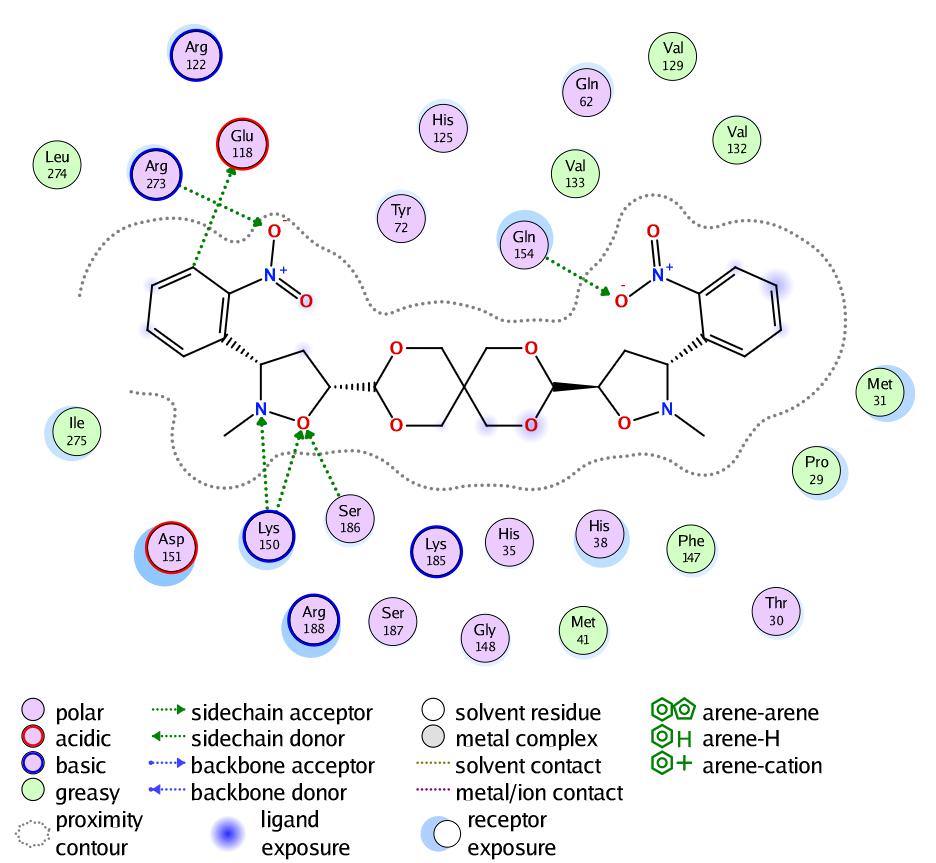**  **4c** | **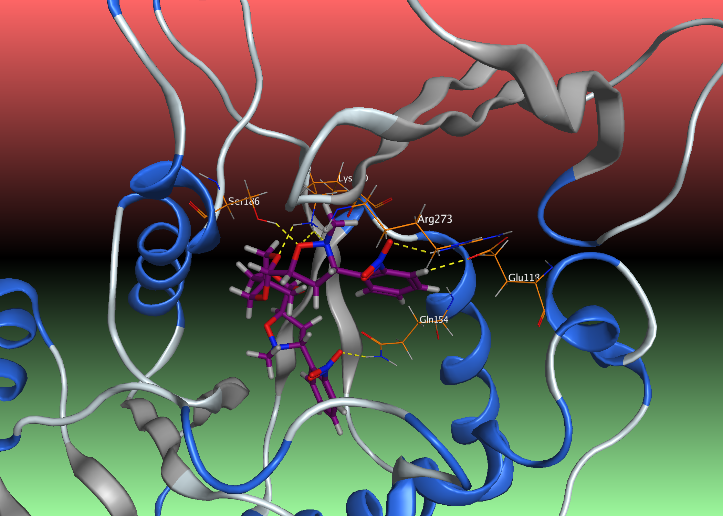**  **4c** |
| 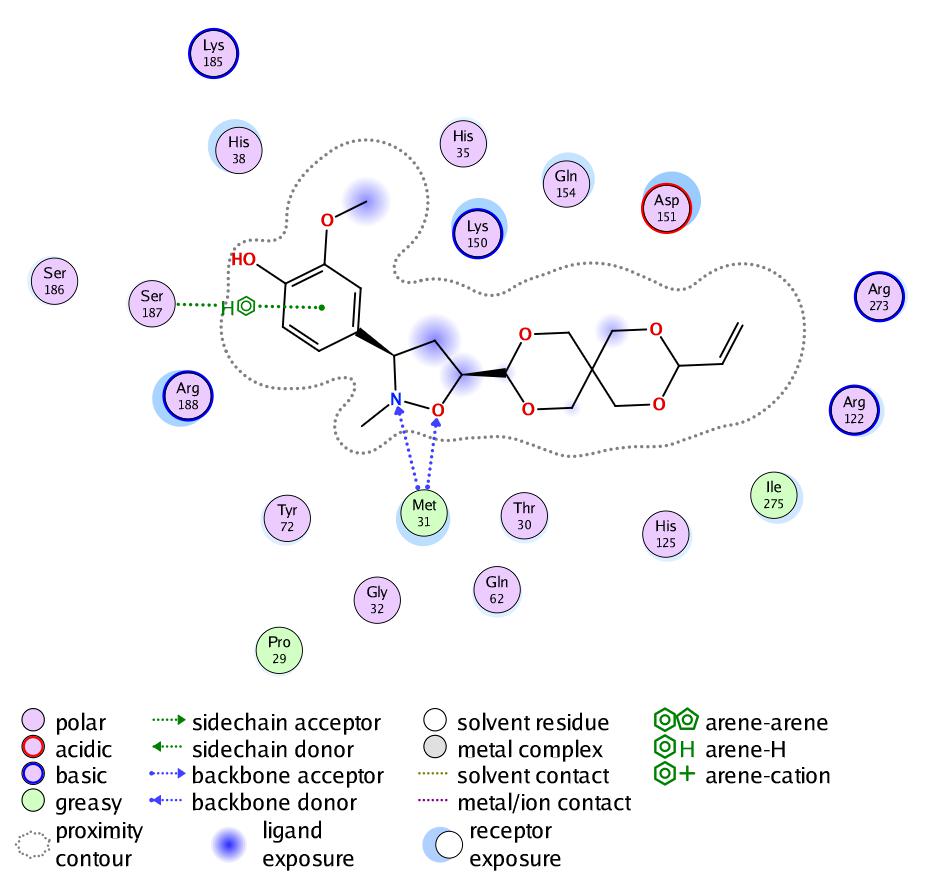  **3e** | 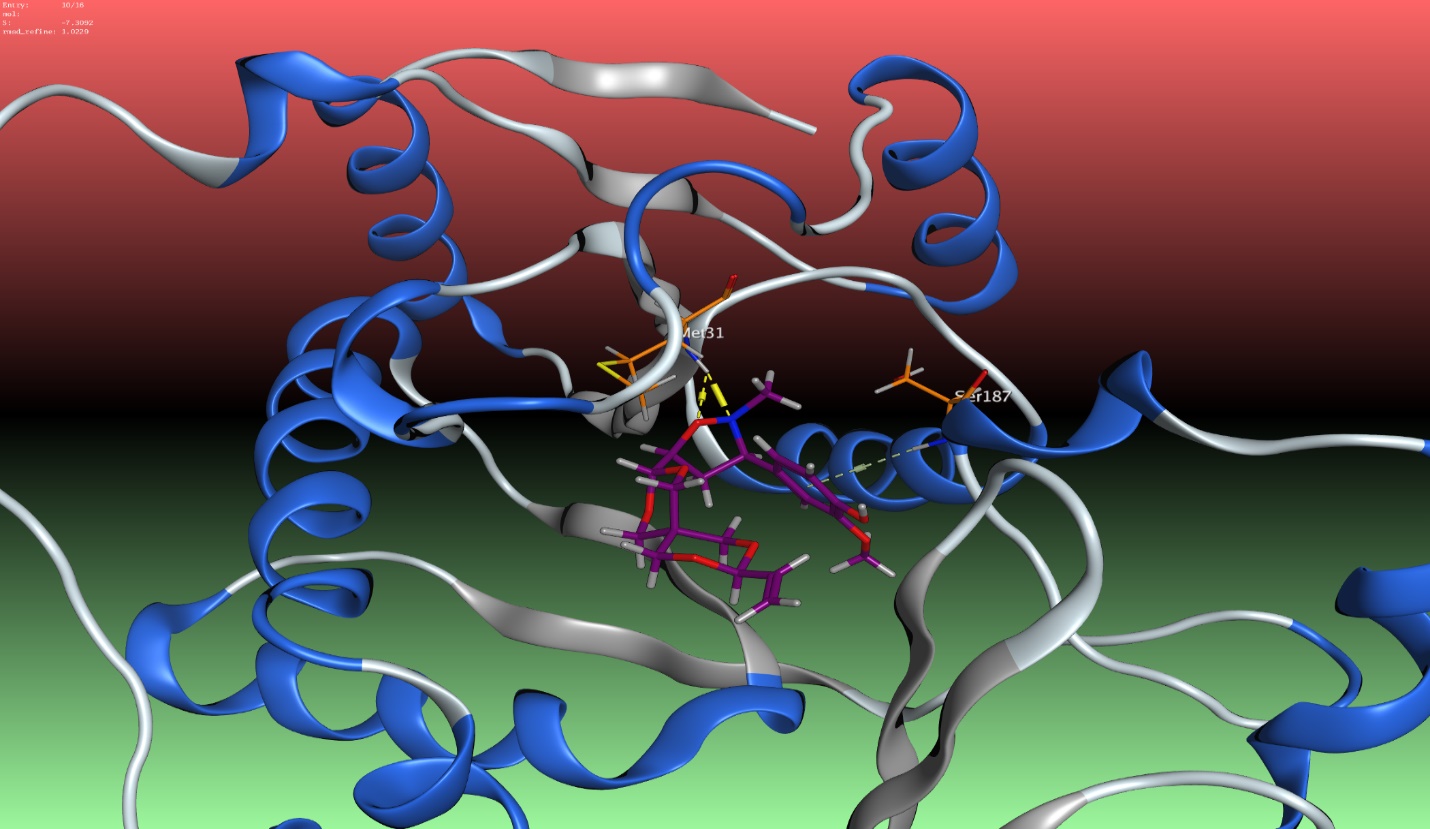    **3e** |
| **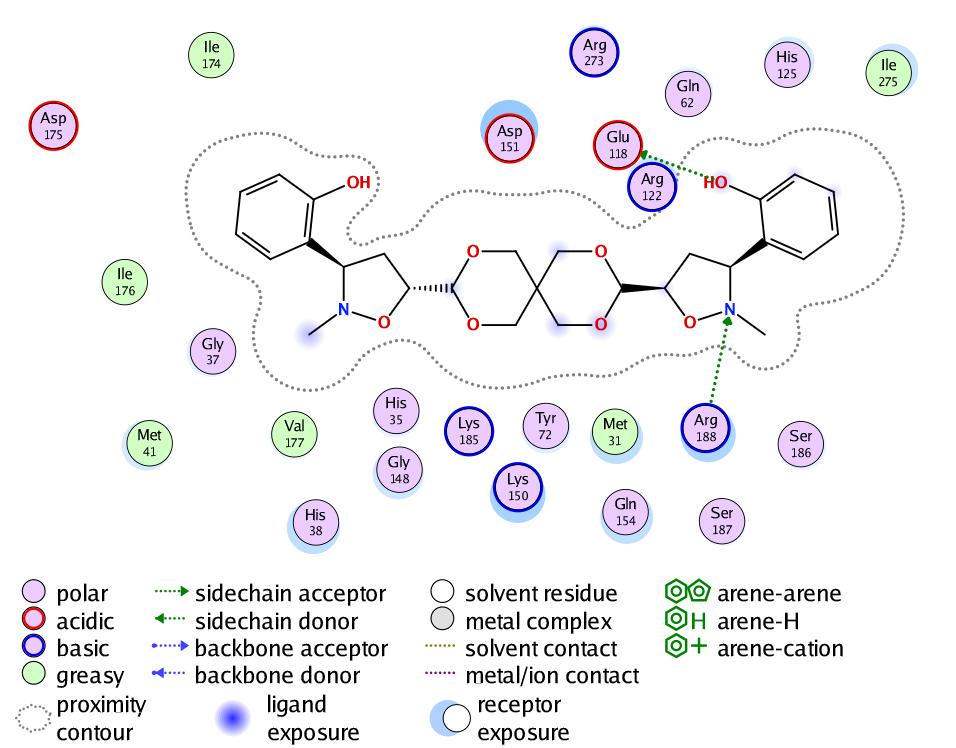**  **4g** | **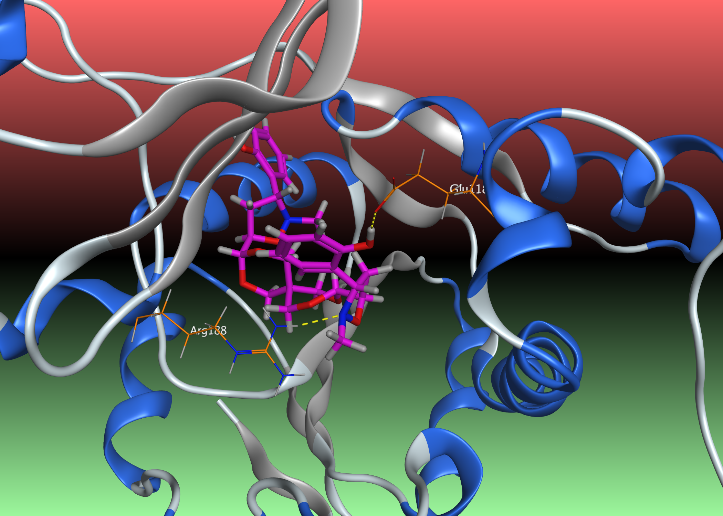**  **4g** |
| **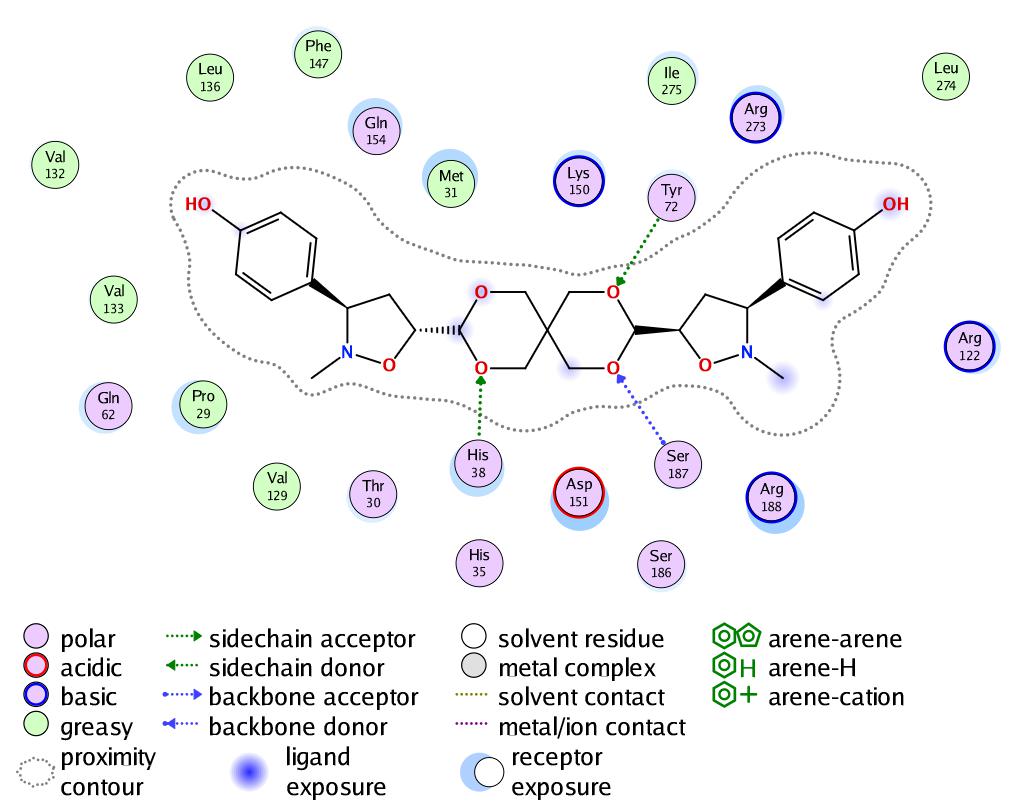**  **4h** | **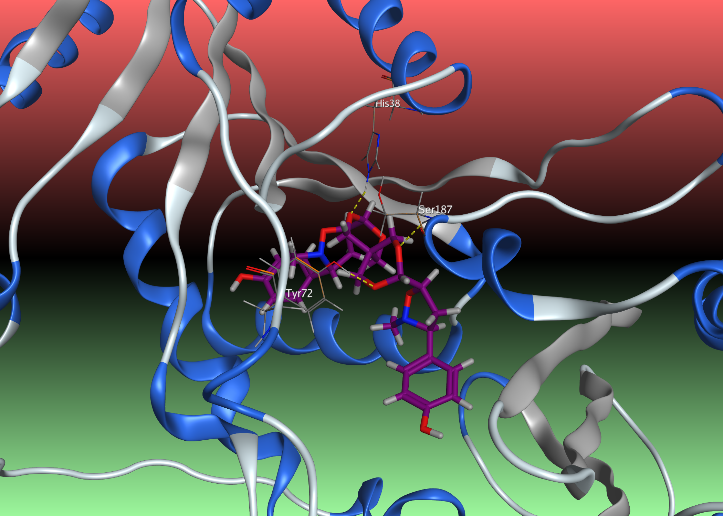**  **4h** |
| **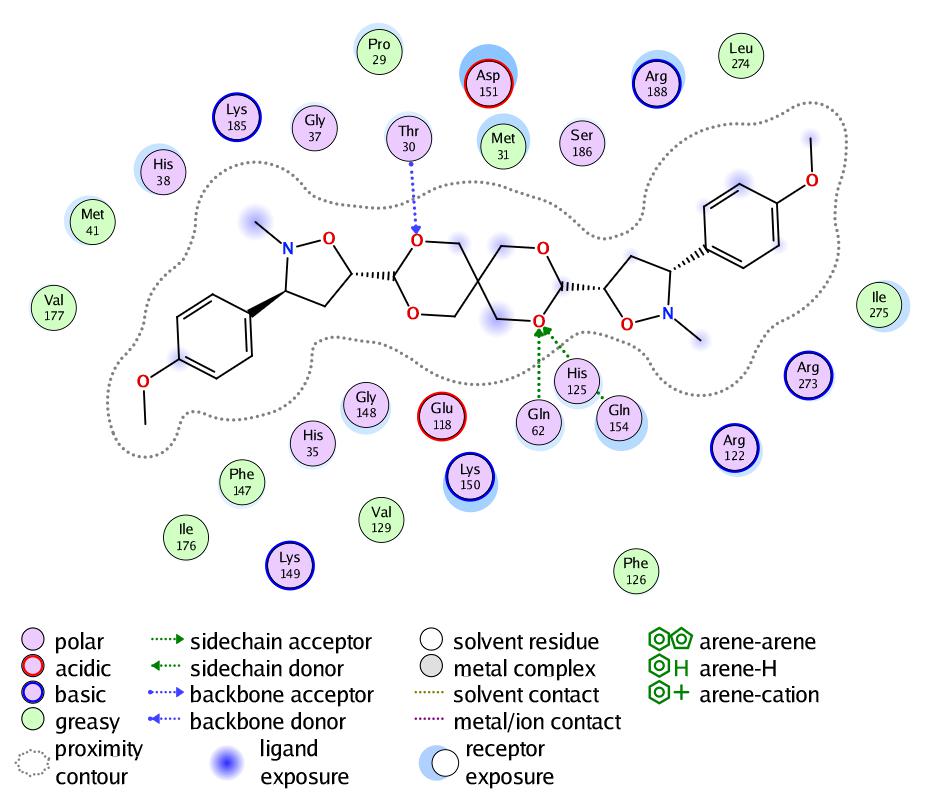**  **4i** | **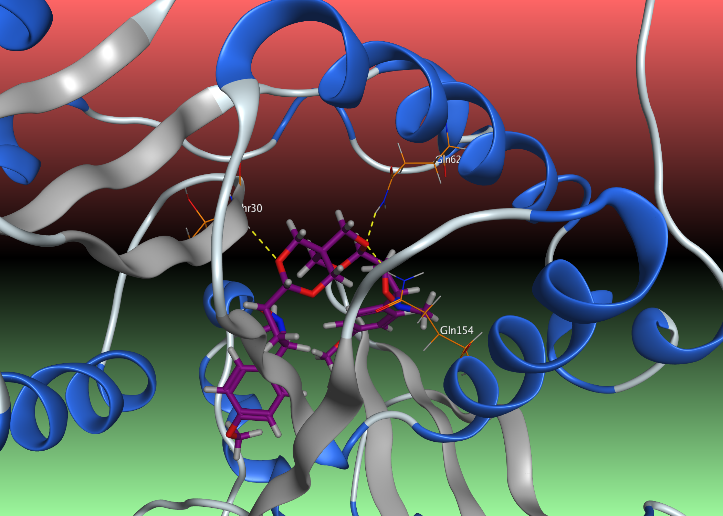**  **4i** |
| **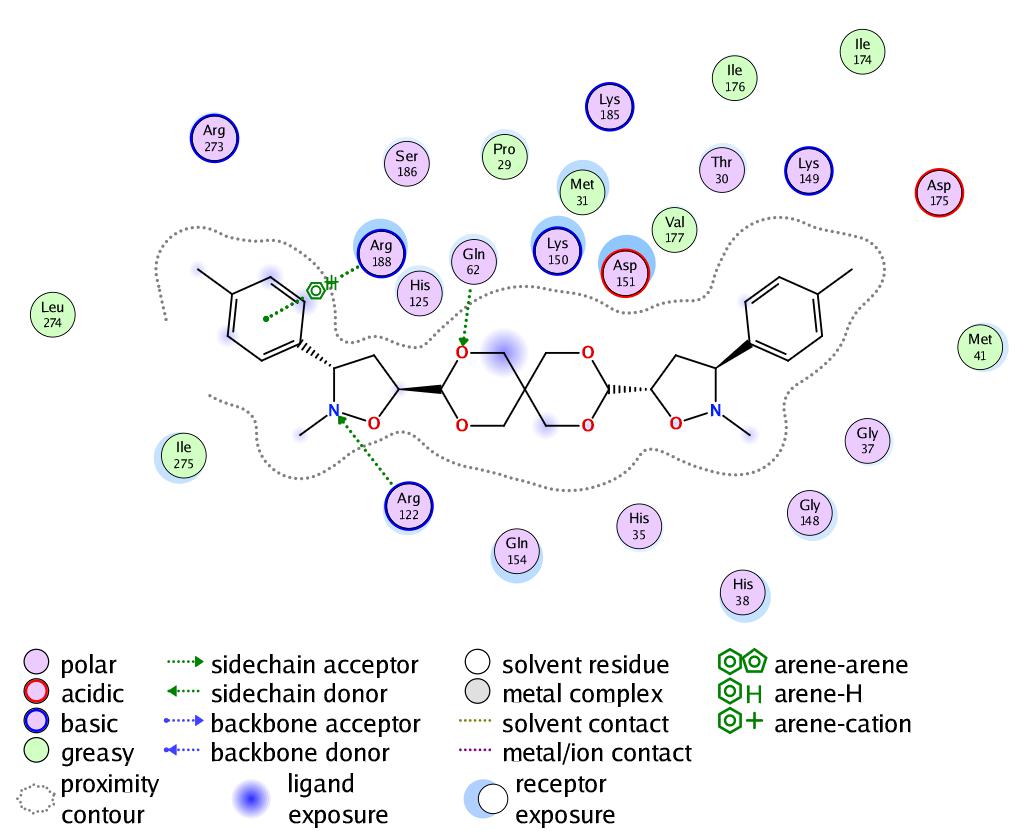**  **4l** | **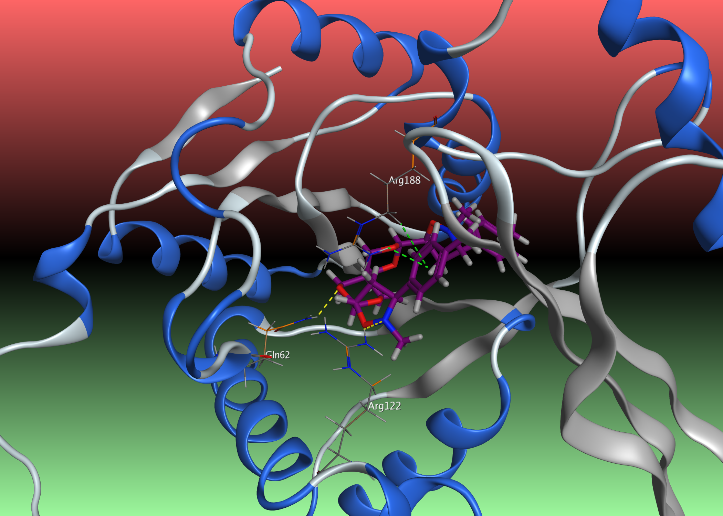**  **4l** |
| **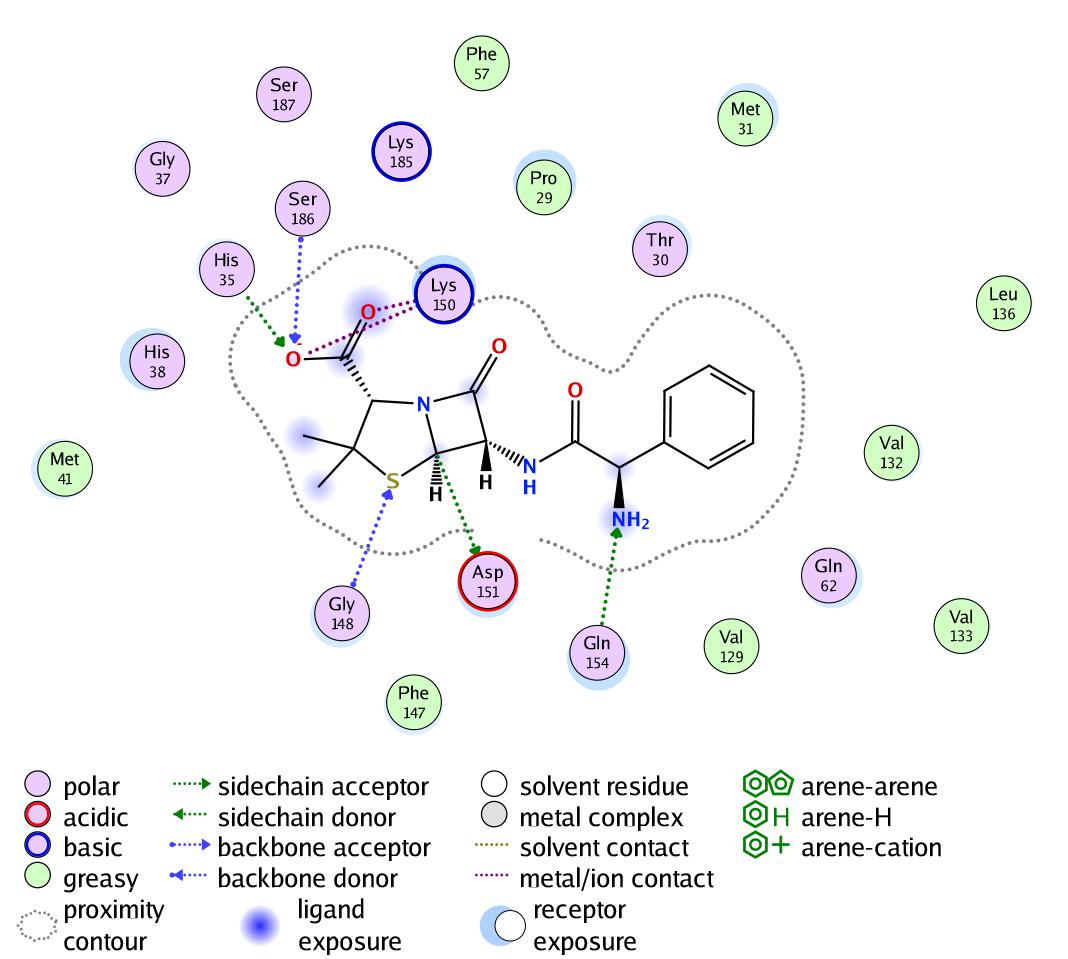**  **Ampicillin** | **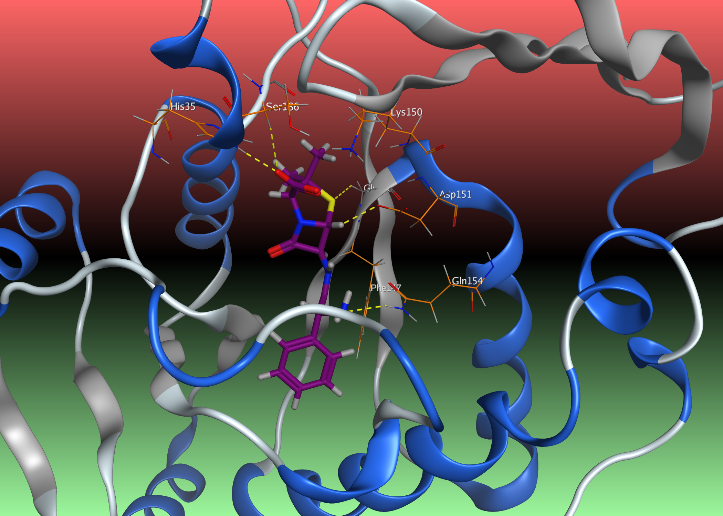**  **Ampicillin** |
| **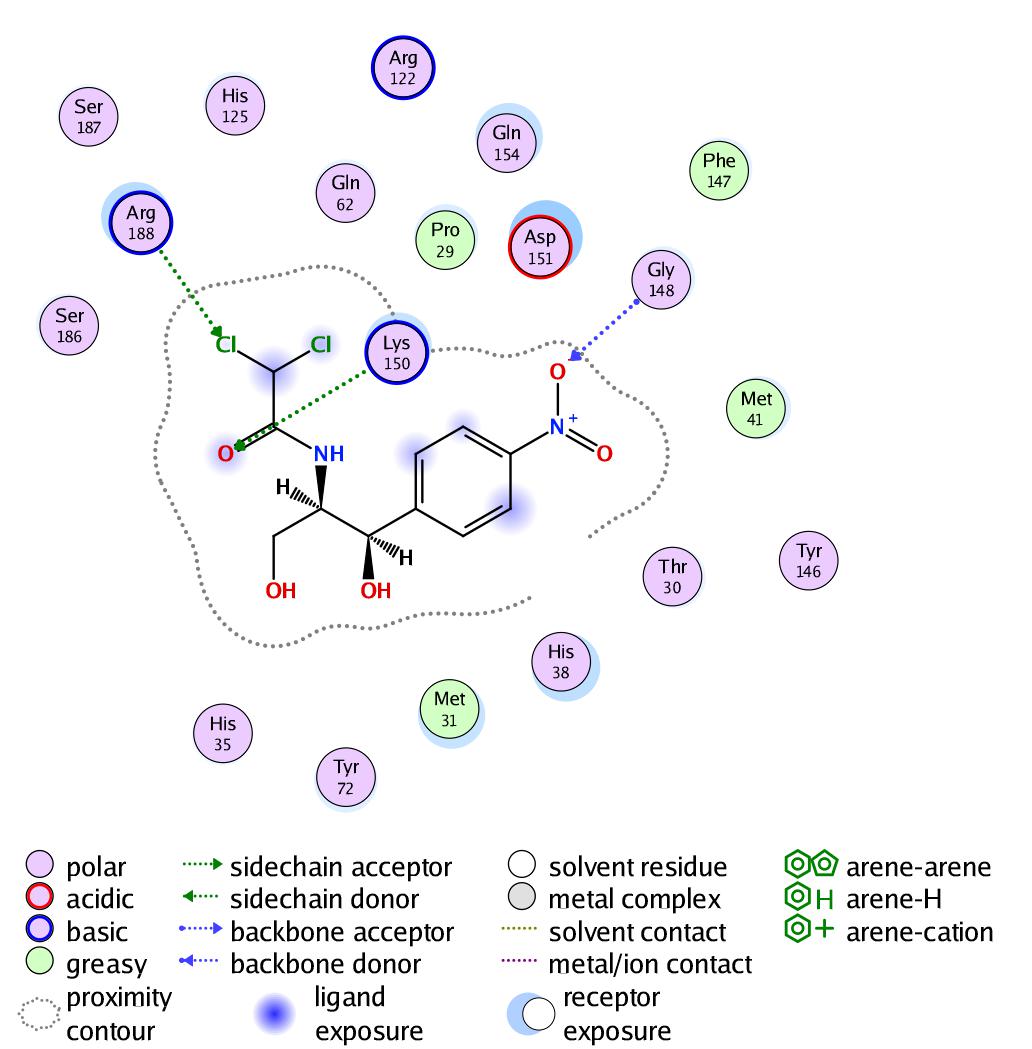**  **Chloramphenicol** | **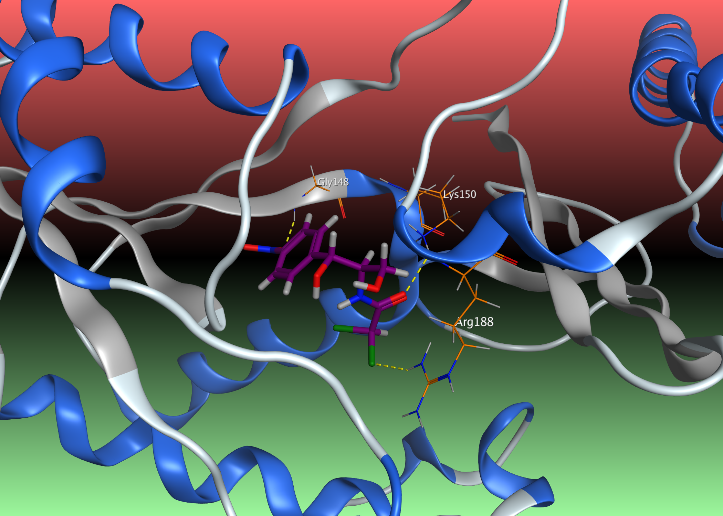**  **Chloramphenicol** |
| **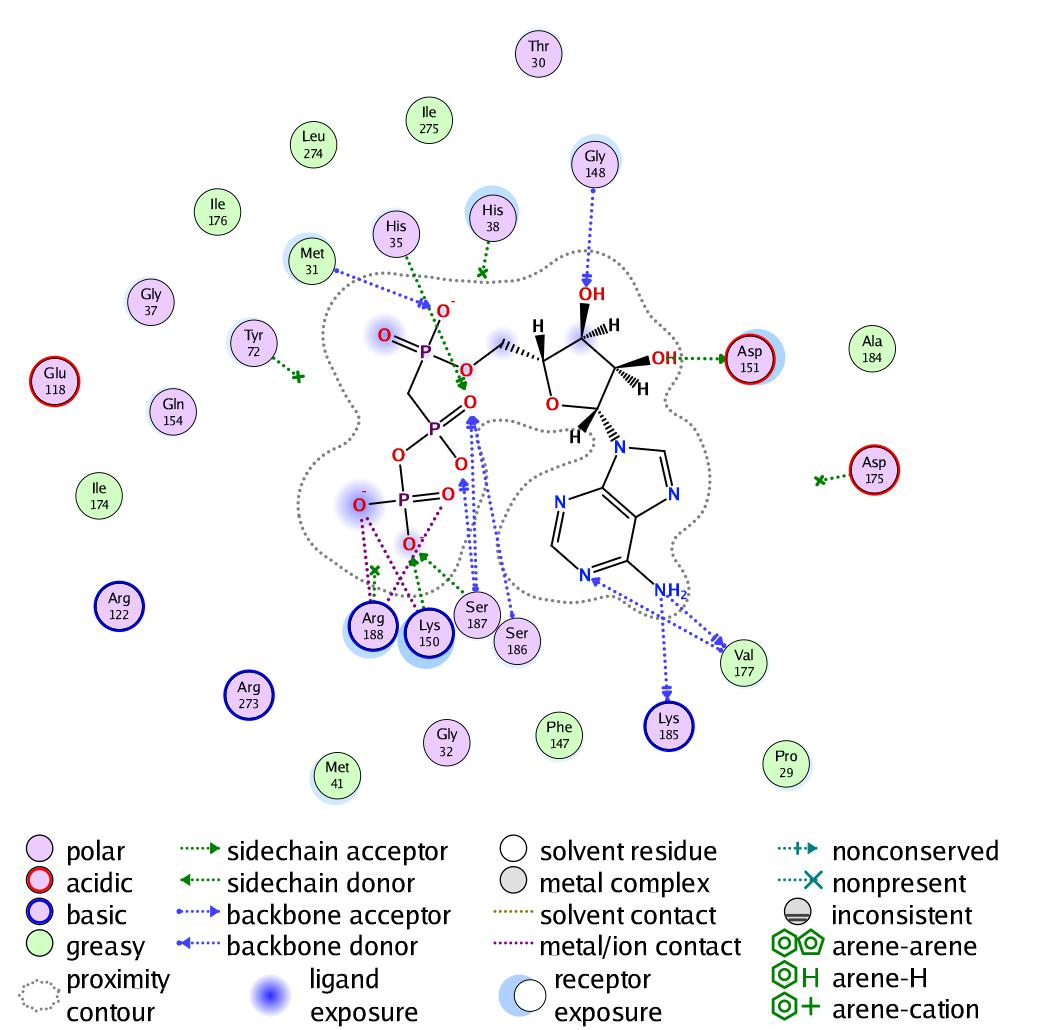**  **Native ligand * (APC)** | **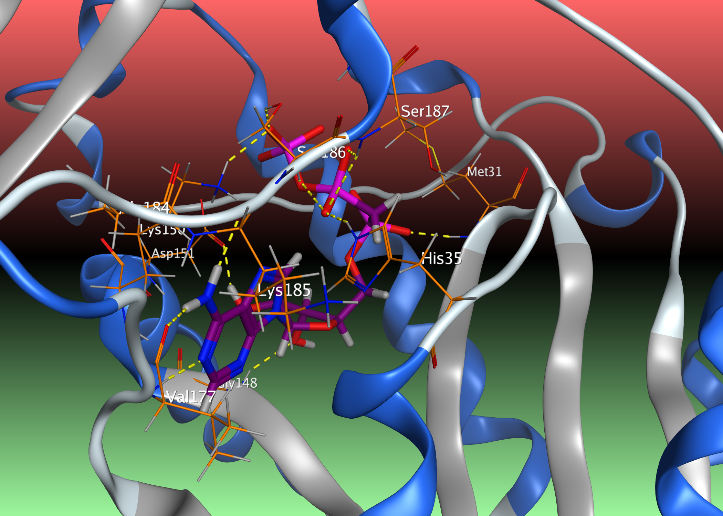**  **Native ligand *(APC)** |

**Figure S50= Figure 7 in the manuscript: Interaction of selected compounds and Ampicillin, Chloramphenicol Native ligand with 2X3F, both 2D (left) and 3D (right) diagrams, is given below with yellow dotted line in the 3D figure represents H-bond**
